# Machine Learning and Metaheuristics Approach for Individual Credit Risk Assessment: A Systematic Literature Review

**DOI:** 10.3390/biomimetics10050326

**Published:** 2025-05-17

**Authors:** Álex Paz, Broderick Crawford, Eric Monfroy, José Barrera-García, Álvaro Peña Fritz, Ricardo Soto, Felipe Cisternas-Caneo, Andrés Yáñez

**Affiliations:** 1 Escuela de Ingeniería en Construcción y Transporte, Pontificia Universidad Católica de Valparaíso, Avenida Brasil 2147, Valparaíso 2362804, Chile; alex.paz@pucv.cl (Á.P.); alvaro.pena@ucv.cl (Á.P.F.); andres.yanez@pucv.cl (A.Y.); 2 Laboratoire d’Étude et de Recherche en Informatique d’Angers (LERIA), Université d’ Angers, 49000 Angers, France; eric.monfroy@univ-angers.fr; 3 Escuela de Ingeniería Informática, Pontificia Universidad Católica de Valparaíso, Avenida Brasil 2241, Valparaíso 2374631, Chile; jose.barrera@pucv.cl (J.B.-G.); ricardo.soto@pucv.cl (R.S.); felipe.cisternas.c@mail.pucv.cl (F.C.-C.)

**Keywords:** individual credit risk assessment, machine learning, metaheuristic, feature selection, bio-inspired algorithms, benchmark datasets, evaluation metrics

## Abstract

Credit risk assessment plays a critical role in financial risk management, focusing on predicting borrower default to minimize losses and ensure compliance. This study systematically reviews 23 empirical articles published between 2019 and 2023, highlighting the integration of machine learning and optimization techniques, particularly bio-inspired metaheuristics, for feature selection in individual credit risk assessment. These nature-inspired algorithms, derived from biological and ecological processes, align with bio-inspired principles by mimicking natural intelligence to solve complex problems in high-dimensional feature spaces. Unlike prior reviews that adopt broader scopes combining corporate, sovereign, and individual contexts, this work focuses exclusively on methodological strategies for individual credit risk. It categorizes the use of machine learning algorithms, feature selection methods, and metaheuristic optimization techniques, including genetic algorithms, particle swarm optimization, and biogeography-based optimization. To strengthen transparency and comparability, this review also synthesizes classification performance metrics—such as accuracy, AUC, F1-score, and recall—reported across benchmark datasets. Although no unified experimental comparison was conducted due to heterogeneity in study protocols, this structured summary reveals consistent trends in algorithm effectiveness and evaluation practices. The review concludes with practical recommendations and outlines future research directions to improve fairness, scalability, and real-time application in credit risk modeling.

## 1. Introduction

Credit risk assessment is critical to financial institutions’ risk management strategies. It involves evaluating the likelihood that a borrower will default on their obligations, thereby protecting the institution from potential financial losses. Accurate prediction and management of credit risk can lead to more informed lending decisions, improved regulatory compliance, and enhanced financial stability [1]. Traditional credit risk assessment methods often relied on centralized data and manual evaluation processes [2]. However, these methods have evolved significantly with the advent of machine learning and artificial intelligence, which allow for the development of more accurate and scalable credit risk models [3,4,5,6]. Among these advancements, particular attention has been given to nature-inspired optimization techniques—such as genetic algorithms, swarm intelligence, and biogeography-based optimization—which reflect a growing interest in computational methods that mimic biological and ecological systems.

**Figure 1:**
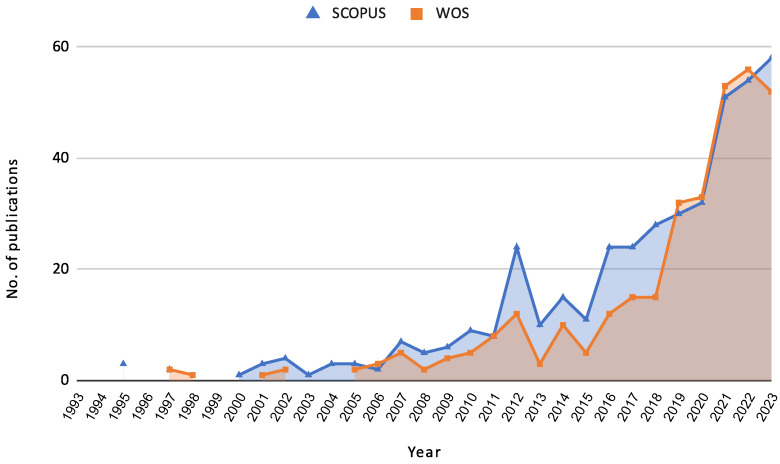
Papers published by years indexed in SCOPUS and WOS from 1993 to 2023.

The growing relevance of credit risk assessment is also reflected in the increasing number of research papers published in this domain. Figure 1 illustrates the evolution of articles published in SCOPUS and Web of Science (WOS) from 1993 to 2023. This trend indicates a heightened interest and ongoing advancements in the field, driven by the integration of advanced machine learning techniques and the need for robust risk management frameworks.

With the integration of machine learning, credit risk models have become more sophisticated, leveraging vast amounts of data to predict borrower behaviour more precisely. This technological evolution has enabled institutions to analyze complex patterns in borrower data, improving the accuracy of predictions and allowing for real-time risk assessment. Machine learning techniques, including logistic regression, decision trees, random forests, support vector machines, and neural networks, provide sophisticated data analysis and prediction tools. These techniques enable the development of models that can handle large datasets and complex relationships among variables. Applying machine learning in credit risk assessment allows for automated decision-making processes, enhancing efficiency and accuracy. Moreover, machine learning models can adapt to new data over time, ensuring that risk assessments remain relevant in dynamic financial environments [2]. Such advancements contribute to a more resilient financial system by enhancing the ability of institutions to manage and mitigate risks associated with lending.

Integrating machine learning techniques has made substantial advancements in credit risk assessment. This subsection summarizes critical literature reviews highlighting this domain’s methodologies, applications, and challenges. The reviews cover various topics, including the performance and interpretability of machine learning models, the potential of federated learning, the factors influencing loan repayment behavior in higher education, and the comparative effectiveness of AI-based methods. These insights are crucial for understanding the current state of research and identifying future directions for improving credit risk assessment models.

- Ref. [2]—This article comprehensively reviews different machine learning models applied in credit risk assessment, focusing on their performance, interpretability, and application in various financial contexts. The study highlights the importance of data preprocessing, feature selection, and optimization techniques in improving model accuracy and reliability.
- Ref. [6]—The paper explores the use of federated learning in credit risk assessment, emphasizing its potential to enhance data privacy and security while maintaining high predictive performance. The authors discuss the challenges and benefits of implementing federated learning in financial institutions but do not specifically focus on feature selection methods.
- Ref. [3]—This comprehensive review covers various AI-based methods for credit risk assessment, evaluating their effectiveness in different scenarios. The authors compare traditional and advanced machine learning techniques, including neural networks and ensemble methods. While the review highlights feature selection and optimization techniques, it does not exclude non-AI methods.
- Ref. [5]—This review focuses on applying machine learning for credit risk prediction, identifying key trends and methodologies in the field. The review highlights the importance of using diverse datasets, feature selection, optimization techniques, and robust evaluation metrics to ensure model reliability and generalizability. However, it includes a variety of statistical methods alongside AI techniques, not exclusively focusing on AI.
- Ref. [4]—This study presents a longitudinal analysis of repayment behaviour in higher education, examining factors that influence loan default rates among students. The findings suggest that demographic and socio-economic variables significantly predict repayment behaviour. However, it does not explicitly focus on feature selection or optimization techniques.

This Systematic Literature Review (SLR) focuses on applying machine learning and optimization techniques for feature selection in individuals’ credit risk assessment. The primary contribution of this SLR is to provide a comprehensive analysis of how machine learning models are utilized to predict credit risk, emphasizing the importance of feature selection methods and optimization techniques. The research addresses several key questions: the types of machine learning algorithms employed, the feature selection methods used, the optimization techniques applied, the datasets and variables commonly utilized, and the evaluation metrics for assessing model performance. By concentrating exclusively on individual credit risk assessment, this review excludes studies focusing on other phenomena such as fraud detection, company valuation, prediction of company bankruptcies, and the impact of macroeconomic changes on credit risk. It also omits research that targets different subjects, like corporate or sovereign debt risk or explores non-predictive credit risk aspects such as sampling method effectiveness or class distribution problems. Additionally, studies that employ only descriptive and analytical statistical methods without integrating artificial intelligence techniques are not considered. Through this focused approach, the SLR aims to fill a critical gap in the literature, providing valuable insights into the most effective machine learning and optimization techniques for enhancing credit risk prediction models for individuals.

Table 1 compares the contributions of the reviewed articles based on eight specific criteria. These criteria were chosen to highlight the unique aspects and strengths of each review, as well as to emphasize the distinctive contributions of this SLR. The criteria are as follows: (1) Focus on Individual Credit Risk Assessment (ICRA), which assesses whether the review targets individual credit risk assessment specifically; (2) Emphasis on Machine Learning (ML), indicating the degree to which the review incorporates machine learning techniques; (3) Emphasis on Feature Selection Techniques (FS), which evaluates the attention given to methods for selecting relevant features in credit risk models; (4) Emphasis on Optimization Techniques (OT), highlighting the focus on methods to optimize model performance; (5) Comprehensive Analysis of Datasets and Variables (D&V), examining the depth of analysis regarding the data sources and critical variables used; (6) Evaluation Metrics (EM), assessing the criteria used to evaluate the effectiveness of credit risk assessment models; (7) Exclusion of Non-Predictive Aspects (EN-PPA), determining if the review excludes non-predictive aspects of credit risk; and (8) Exclusion of Non-AI Techniques (EN-AIT), indicating whether the review solely considers AI-based techniques. This detailed comparison clearly explains how each review contributes to credit risk assessment.

To summarize, this SLR makes a distinctive and significant contribution to credit risk assessment by focusing exclusively on individual credit risk. In contrast to broader reviews such as [3,5], which encompass various financial contexts and include both predictive and non-predictive approaches, our review provides a more targeted analysis centered on machine learning-based predictive modelling for individuals. Furthermore, we place particular emphasis on feature selection and optimization techniques—two aspects that are often mentioned but rarely explored in depth in prior work. Additionally, our review excludes non-AI and descriptive statistical approaches, maintaining a strict focus on advanced machine learning techniques. This comprehensive and focused approach addresses a relevant gap in the literature and contributes to a more nuanced understanding of the state of the art in individual credit risk prediction.

Although previous reviews have significantly contributed to understanding machine learning applications in credit risk, they tend to address the field from a generalist perspective. Most integrate studies across corporate, sovereign, and individual domains, and only a few explicitly differentiate among them. As a result, the specific methodological challenges and modelling strategies unique to individual credit risk are often overlooked or diluted within broader discussions. Moreover, prior reviews that mention feature selection or optimization techniques typically do so superficially, without comparative analysis or synthesis of their practical implementation and impact on predictive performance.

To address these limitations, this SLR was designed around five research questions that directly target the overlooked aspects identified in previous work. Table 1 offers a structured comparison, highlighting how our review differs in scope, depth, and methodological rigor. By narrowing the focus and deepening the analysis, this review provides an updated and more actionable perspective on how machine learning and optimization can be applied to improve individual credit risk modelling.

To support this objective, we conducted a systematic review following PRISMA guidelines, identifying and analyzing 23 empirical studies published between 2019 and 2023 that focus on individual credit risk assessment using machine learning techniques. In addition, this review organizes and synthesizes the classification performance metrics reported in the reviewed studies, particularly for benchmark datasets. While no direct empirical comparison is conducted due to the variability in experimental conditions, this synthesis helps identify patterns in reported model effectiveness and metric usage across studies.

The remainder of this document is structured as follows: Section 2 presents the applied methodology and details the research questions. The research questions proposed in Section 2 are answered in Section 3. This section begins with a brief characterization of the reviewed documents, followed by the results of the extracted data for each guiding research question. Finally, Section 4 discusses the findings of the research and suggests directions for future work.

## 2. Methodology

The SLR follows the PRISMA framework [7] and the Guidelines for performing Systematic Literature Reviews in Software Engineering [8]. The review is structured into three main phases: Planning, Conducting, and Reporting.

In the planning phase, the need for a systematic review in credit risk assessment was identified, highlighting its importance for financial decision-making and risk management. Clear and focused research questions were specified to guide the review and ensure a targeted and relevant investigation of the literature. A detailed review protocol was developed, outlining the methods and standards for conducting the review, including search strategies, inclusion and exclusion criteria, and data extraction procedures.

The conducting phase involved identifying relevant research through comprehensive searches in the SCOPUS and WOS databases using predefined keywords and search strings. Studies were screened and selected based on predefined inclusion and exclusion criteria to ensure relevance and quality. To enhance the reliability of the selection process, the screening and eligibility phases were carried out independently by multiple reviewers. Each reviewer assessed the titles, abstracts, and full texts according to the established criteria. Discrepancies or disagreements regarding the inclusion of specific studies were resolved through discussion and consensus among the reviewers. This procedure helped mitigate selection bias and strengthened the internal validity of the review process.

Subsequently, the reliability and validity of the selected studies were evaluated to ensure robust conclusions. Data extraction followed a systematic and structured procedure to maintain consistency and comprehensiveness. The extracted data were then analyzed and synthesized to identify trends, research gaps, and key findings within the literature.

Addition, to further support our analysis, we extracted the classification performance metrics reported in the included studies for benchmark datasets only. Metrics such as accuracy, AUC, F1-score, precision, and recall were systematically recorded when available. No attempt was made to standardize or replicate the experimental conditions; the aim was to provide a structured overview of reported results and to enhance the transparency and comparability of evaluation practices across the literature.

In the reporting phase, the flow of information through the different stages of the review was documented, including the number of records identified, included, and excluded, as well as the reasons for exclusion. The findings were organized and presented in a structured and coherent manner in the Findings Section 3. This section first characterizes the selected documents and then presents the results related to each research question. The characterization includes aspects such as the number of publications per year, distribution of publications by editorial groups, journal subject area trends, documents by publication title or journal, and the citation report of the reviewed documents. The subsequent sections present the data extracted in response to each research question.

**Figure 2:**
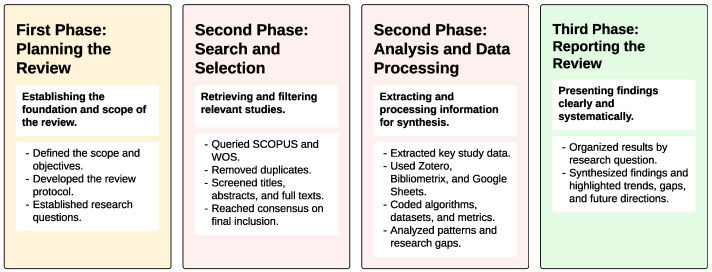
Visual Summary of the SLR Methodology: Phases, Actions, and Tools.

To complement the textual description above, Figure 2 provides a graphical summary of the methodological workflow followed in this systematic literature review. The figure outlines the key activities, decisions, tools, and outputs associated with each phase of the process—planning, search and selection, data processing, and reporting. This visual representation is intended to offer a concise and accessible overview of how the review was conducted from end to end.

The review is guided by the following research questions, which aim to provide a comprehensive understanding of the application of machine learning techniques in credit risk assessment from multiple perspectives (Table 2).

The search strategy was designed to ensure a comprehensive identification of relevant studies. This systematic review relied exclusively on SCOPUS and WoS, as the research team considered them to provide extensive coverage of peer-reviewed literature, advanced filtering capabilities, and robust citation indexing. These databases are widely regarded as gold standards for systematic reviews across technical and interdisciplinary domains, particularly in finance, computer science, and engineering. This choice aligns with established guidelines for systematic reviews in computing and engineering domains, which recommend the use of reputable, peer-reviewed sources [8].

In contrast, databases such as Google Scholar were excluded due to their limited filtering options, opaque indexing policies, and inclusion of non-peer-reviewed content. IEEE Xplore and PubMed were also excluded, as their primary focus on engineering and biomedical literature, respectively, was deemed misaligned with the study’s specific scope on individual credit risk assessment using machine learning. Based on these considerations, the research team determined that SCOPUS and WoS were both sufficient and appropriate to ensure the quality and relevance of the selected studies.

Search terms were developed to capture the fundamental concepts of credit risk assessment and machine learning. The search terms are structured to include synonyms and related concepts to ensure a comprehensive search. The search was conducted at two levels: a focused search using keywords like *Credit risk assessment*, *Repayment Behavior*, *Loan repayment behaviour*, *Loan Payment*, *Payment Behavior*, *Debt repayment*, *Borrower payment habits*, *Loan default predictors*, and combining them with *Machine Learning*, *Artificial intelligence*, *Predictive Model*, *Classification model*, and *Prediction*.

Specific inclusion and exclusion criteria were applied to ensure the selected studies’ relevance and quality. Inclusion criteria encompassed studies published between 2019 and 2023, articles in English, and those appearing in indexed journals. Exclusion criteria included studies on phenomena unrelated to credit risk assessment, such as fraud detection, company valuation, prediction of company bankruptcies, and studies investigating subjects other than individual credit risk. Studies not addressing predictive aspects of credit risk or those using descriptive and analytical statistical methods instead of AI techniques were also excluded.

The configured queries for each database are as follows:

**SCOPUS:** *(TITLE-ABS-KEY (“Credit risk assessment” OR “Repayment Behavior” OR “Loan repayment behavior” OR “Loan Payment” OR “Payment Behavior” OR “Debt repayment” OR “Borrower payment habits” OR “Loan default predictors”) AND TITLE-ABS-KEY (“Machine Learning” OR “Artificial intelligence” OR “Predictive Model” OR “Classification model” OR “Prediction”)) AND PUBYEAR > 2018 AND PUBYEAR < 2024 AND (LIMIT-TO (DOCTYPE “ar”)) AND (LIMIT-TO (LANGUAGE “English”))*

**WOS:** *(TS=(”Credit risk assessment” OR “Repayment Behavior” OR “Loan repayment behavior” OR “Loan Payment” OR “Payment Behavior” OR “Debt repayment” OR “Borrower payment habits” OR “Loan default predictors”) AND TS=(”Machine Learning” OR “Artificial intelligence” OR “Predictive Model” OR “Classification model” OR “Prediction”)) AND (PY==(”2023” OR “2022” OR “2021” OR “2020” OR “2019”) AND DT==(”ARTICLE”) AND LA==(”ENGLISH”)*

We aim to comprehensively cover the literature relevant to our research questions by systematically applying these search strategies. This structured approach will help synthesize a wide range of information and draw informed conclusions.

The study selection process for this SLR follows the PRISMA framework to ensure a rigorous and transparent methodology. Initially, the search results from SCOPUS and WOS databases were imported into a reference management tool to standardize the fields provided by the two databases and facilitate the removal of duplicates. The screening process involved two stages. First, the exported bibliographical data fields were reviewed to ensure the documents met the inclusion criteria. In the second stage, titles and abstracts were analyzed based on the exclusion criteria to exclude studies not meeting the predefined inclusion criteria. Studies were excluded if they focused on phenomena unrelated to credit risk assessment, investigated subjects other than individual credit risk, did not address predictive aspects of credit risk, or used descriptive and analytical statistical methods instead of AI techniques. Following this, during the eligibility assessment, a full-text screening was conducted to assess the relevance of the remaining studies based on evaluating the full texts to ensure they met the criteria related to the research questions. In this stage, articles pertinent to SLRs, reviews, or surveys were excluded.

To enhance the reliability of the study selection process, all screening and eligibility steps were conducted independently by multiple reviewers. Each reviewer applied the predefined inclusion and exclusion criteria to the titles, abstracts, and full texts. Disagreements or inconsistencies regarding the inclusion of specific studies were resolved through collaborative discussion until consensus was achieved. This approach helped mitigate selection bias and reinforced the internal validity of the review methodology.

**Figure 3:**
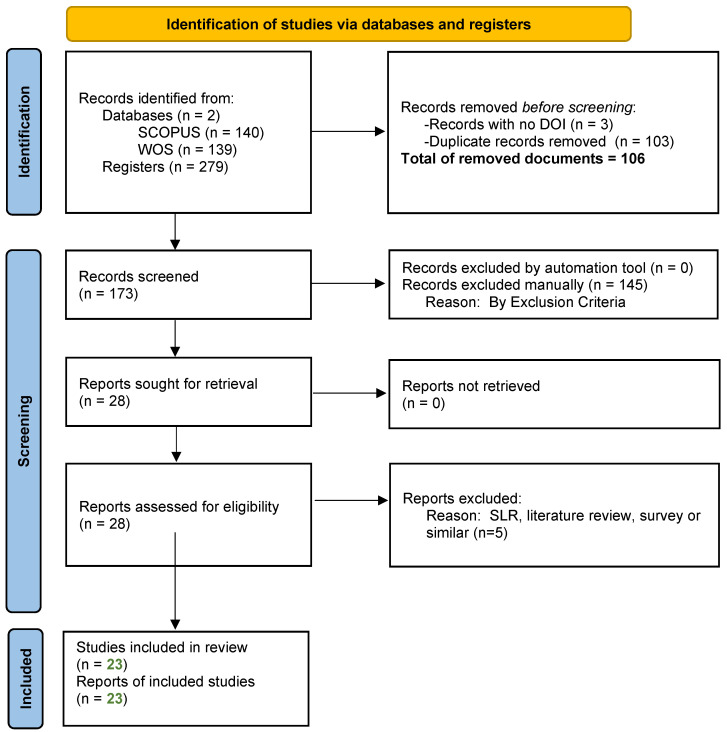
PRISMA Flow Diagram.

The final inclusion of studies was based on their relevance to the research questions and the quality of their methodologies. The PRISMA flow diagram (Figure 3) maps the number of records identified, included, and excluded at each stage and the reasons for exclusions.

To support the management and analysis of the included studies, several tools were employed. Zotero (version 6.0.35) was used for reference management and citation organization. R (version 3.5.1, 2018-07-02) and RStudio (version 2024.09.0 Build 375, “Cranberry Hibiscus” release), along with the Bibliometrix package (version 4.1.4), were utilized to conduct the bibliometric analysis and generate key visualizations. Additionally, Google Sheets was used to manually register the extracted data from each study and to produce supporting summary charts for internal tracking and synthesis.

## 3. Findings

This section presents the key findings from the reviewed articles, focusing on the methodologies and techniques employed in credit risk assessment. It is organized into several subsections that cover the Characteristics of Reviewed Documents, Machine Learning Techniques, Feature Selection Methods, Optimization Techniques (Metaheuristics), Applications in Practice, Datasets and Variables, and Evaluation Metrics.

### 3.1. Characterization of Reviewed Documents

This section presents a characterization of the reviewed documents, providing insights into publication trends, distribution across editorial groups, subject areas, and citation metrics.

**Figure 4:**
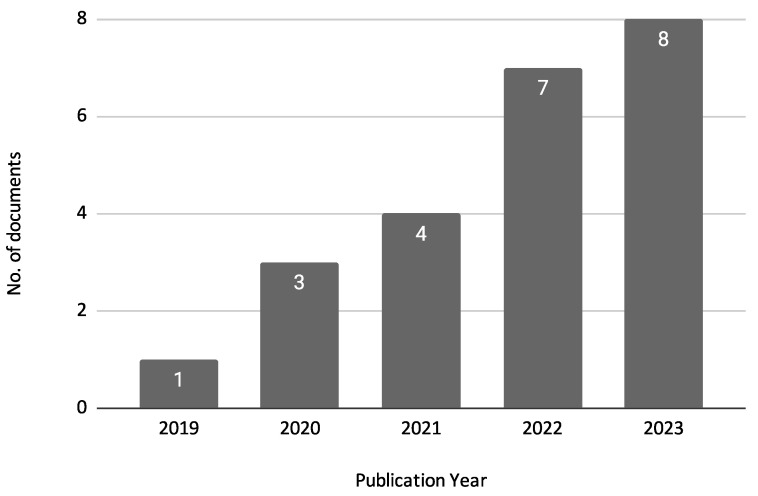
Number of Publications by Year.

Figure 4 shows the annual distribution of the reviewed documents. This analysis helps to identify trends in research activity over time, highlighting periods of increased focus on credit risk assessment using machine learning techniques. The figure indicates that there has been a growing interest in this research area, particularly in recent years. In 2019, there was only one publication, but this number increased to 3 in 2020 and 4 in 2021. The upward trend continues with seven publications in 2022 and peaks in 2023 with eight publications. This steady increase in publications reflects the escalating importance and application of machine learning techniques in credit risk assessment.

**Figure 5:**
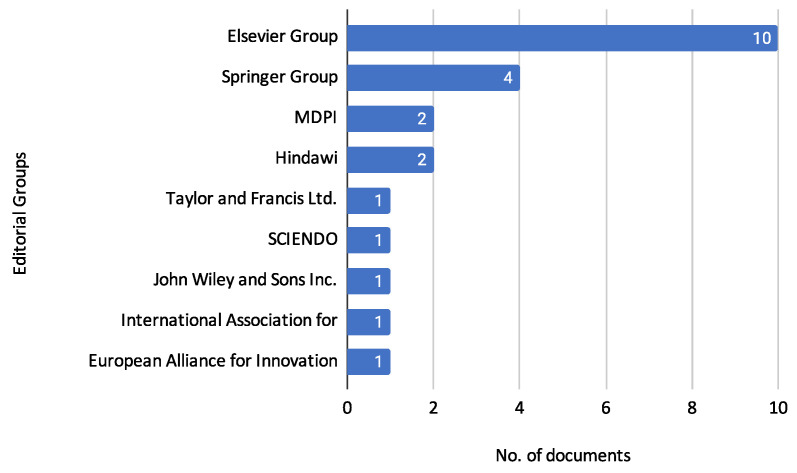
Distribution of Publications Across Editorial Groups.

The observed increase in publications reinforces the growing recognition and importance of machine learning techniques in credit risk assessment. Figure 5 presents the distribution of publications across various editorial groups to understand further where these studies are being published. This analysis provides an understanding of the preferred publication outlets for research in this domain, indicating the diversity of journals and conferences that feature studies on credit risk assessment.

The Elsevier Group is the most prominent, publishing ten reviewed documents. The journals under this group include *Applied Soft Computing*, *Computers and Education: Artificial Intelligence*, *Computers and Operations Research*, *Expert Systems with Applications*, *Information Sciences*, *Journal of Computational and Applied Mathematics*, and *Research in International Business and Finance*.

The Springer Group follows with four documents, published in *Innovations in Systems and Software Engineering*, *International Journal of Information Technology (Singapore)*, *Multimedia Tools and Applications*, and *SN Computer Science*.

MDPI has contributed two documents to the reviewed set, appearing in *Entropy* and the *Journal of Risk and Financial Management*.

Similarly, Hindawi has published two documents, specifically in *Computational Intelligence and Neuroscience* and *Wireless Communications and Mobile Computing*.

Other editorial groups have published fewer documents: Taylor and Francis Ltd. published 1 document in *Fuzzy Information and Engineering*; SCIENDO published 1 document in the *Journal of Applied Mathematics Statistics and Informatics*; John Wiley and Sons Inc. published 1 document in *Intelligent Systems in Accounting, Finance and Management*; The International Association for Educators and Researchers (IAER) published 1 document in the *Annals of Emerging Technologies in Computing*; The European Alliance for Innovation published 1 document in *EAI Endorsed Transactions on Scalable Information Systems*.

This distribution illustrates the broad range of editorial groups and journals actively publishing research in credit risk assessment, reflecting its interdisciplinary nature and wide-reaching relevance.

Next, we examine the specific subject areas of the journals where these studies are published. The following figure shows the trend of journal subject areas over the years, demonstrating how credit risk assessment research intersects with various academic fields such as computer science, engineering, mathematics, and more.

**Figure 6:**
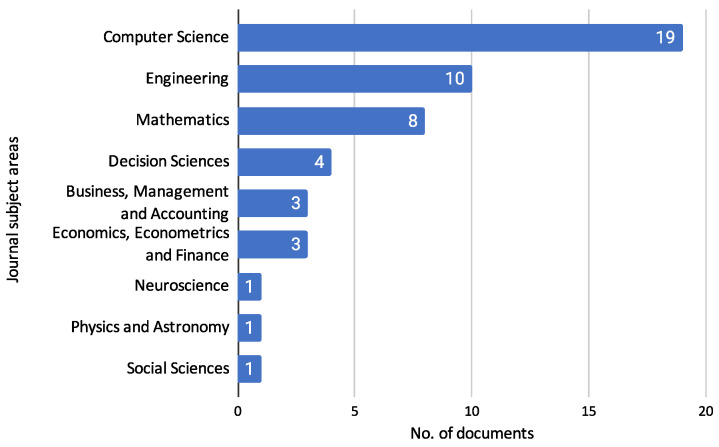
Journal Subject Areas Trend.

Figure 6 shows the distribution of reviewed documents across different journal subject areas. This analysis highlights the interdisciplinary nature of the research, demonstrating how credit risk assessment intersects with various fields such as finance, computer science, and engineering.

The Figure 6 indicates that most reviewed documents are published in journals categorized under Computer Science, with 19 documents. This is followed by Engineering, which accounts for ten papers, and Mathematics, with eight documents. Four documents represent Decision Sciences. Other subject areas include Business, Management and Accounting, Economics, Econometrics, and Finance, each with three documents. Additionally, there is 1 document each in Neuroscience, Physics and Astronomy, and Social Sciences. It is important to note that journals can be indexed under multiple subject areas in SCOPUS, reflecting the multifaceted nature of their content. Moreover, although SCIENDO is not indexed in SCOPUS, the journals published under this editorial group were categorized according to the SCOPUS classification system based on their aim and scope. This comprehensive categorization underscores credit risk assessment research’s diverse and interdisciplinary landscape, spanning various scientific and practical domains.

To further illustrate this diversity, Table 3 lists the Journal Titles of the 23 reviewed articles and the corresponding documents. This overview highlights the variety of journals where research on credit risk assessment has been published during this SLR.

Table 3 shows that the journal *Expert Systems with Applications* has the highest number of reviewed documents, with three publications. Several journals, including *Applied Soft Computing*, have two publications each. In contrast, many others have one publication each, such as *Wireless Communications and Mobile Computing* and *SN Computer Science*. This diversity illustrates the wide range of platforms that publish research on credit risk assessment, highlighting the interdisciplinary appeal and significance of this research area.

**Figure 7:**
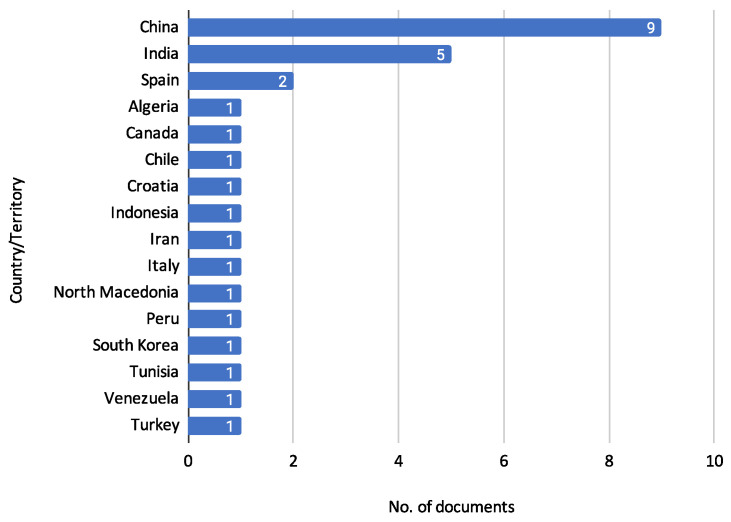
Documents by country or territory.

In addition to analyzing Journal Titles, it is also essential to consider the geographic distribution of the reviewed documents. Understanding the countries and territories where this research is conducted provides insights into the global reach and collaboration in credit risk assessment. Figure 7 describes the distribution of documents concerning the country affiliation of the authors.

Figure 7 presents the author’s affiliation, country, or territory distribution of reviewed documents. China leads with nine documents [11,13,14,16,19,24,28,29,30], reflecting a significant contribution to the research in credit risk assessment. India follows with five documents [12,17,21,22,23], indicating substantial interest and research activity in this area. Spain has two documents [10,15], while Algeria [15], Canada [10], Chile [10], Croatia [26], Indonesia [23], Iran [25], Italy [31], North Macedonia [18], Peru [27], South Korea [22], Tunisia [9], Venezuela [27], and Turkey [20] each have one document. This distribution reinforces the global interest and collaborative efforts in advancing the field of credit risk assessment.

To further understand the impact of these publications, Table 4 presents the citation metrics for the reviewed documents. This analysis provides insights into the impact and recognition of these studies within the academic community, as indicated by the number of times other researchers have cited each document.

The document by [12] has the highest citation count, with 113 citations, reflecting its significant influence and recognition within the field. It is followed by the work of [9], which has been cited 54 times. Additionally, the study by [19] has accumulated 27 citations, while [11] has received 26 citations.

Other documents with notable citation counts include Ref. [13] with 19 citations, ref. [28] with 16 citations, and ref. [24] with 15 citations. Ref. [22] has been cited 14 times, while ref. [18] has received 11 citations. Ref. [21] has 10 citations, and ref. [26] has 7 citations.

Several documents have been cited fewer times, including ref. [25] with 5 citations, refs. [15,16] each with 4 citations, and refs. [10,14,23,27] each with 3 citations. Ref. [30] has 2 citations, and refs. [20,29,31] each have 1 citation. Finally, Ref. [17] has not been cited yet.

This distribution of citations emphasizes the varying levels of impact that different studies have had in the field. Some documents are highly influential, while others are less recognized but still contribute valuable insights.

### 3.2. Machine Learning in Credit Assessment

The methodologies employed in credit risk assessment are varied and sophisticated. They harness the capabilities of machine learning to predict borrower behaviour accurately.

Machine learning techniques are usually categorized into three main classes based on their learning approach and the purpose of the algorithm. These categories are described in the work of [32], where the authors distinguish between supervised learning (SL), unsupervised learning (UL), and reinforcement learning (RL).

- **Supervised Learning (SL):** This category includes traditional algorithms such as Logistic Regression (LR), Decision Trees (DT), Random Forest (RF), Support Vector Machines (SVM), and Gradient Boosting Machines (GBM). These methods require labelled data to train models to predict the likelihood of default.
- **Unsupervised Learning (UL):** Techniques such as clustering and anomaly detection fall under this category. Methods like K-means and Principal Component Analysis (PCA) are used to identify patterns and outliers in the data without the need for labelled training samples.
- **Reinforcement Learning (RL):** RL involves training algorithms based on the reward feedback from their actions, optimizing long-term performance through trial and error.

**Figure 8:**
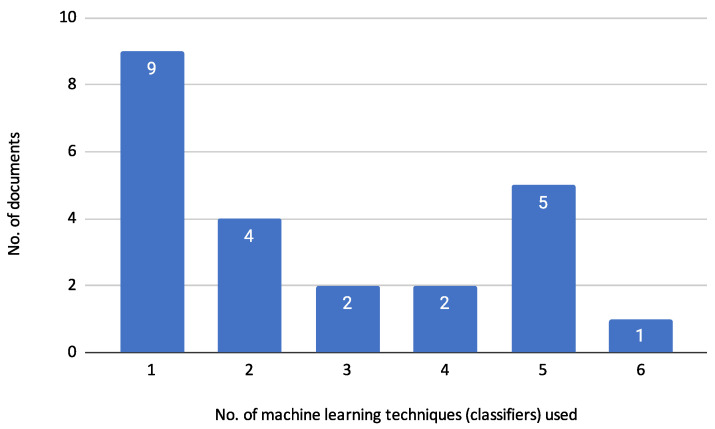
Frequency of Classifier Use Across Documents.

The reviewed articles employed various machine learning algorithms for credit risk assessment, primarily focusing on classification tasks under the Supervised Learning (SL) approach. These tasks involve using labelled data to train models that predict the likelihood of default. Figure 8 illustrates the distribution of the number of machine learning classifiers used across the reviewed documents. The figure shows that the majority of the documents (9) employed only a single classifier for their analysis [9,10,13,14,17,22,24,27,30]. Four documents used two classifiers [16,19,21,23], while two documents used three classifiers [15,20]. Similarly, two documents utilized four classifiers [12,29]. Five documents employed five classifiers [18,25,26,28,31], and one document used six classifiers [11]. This distribution indicates a tendency among researchers to use a limited number of classifiers, with a significant proportion opting for just one or two classifiers in their studies. The variation in the number of classifiers used also suggests different approaches and levels of complexity in the credit risk assessment models presented in the reviewed articles.

#### 3.2.1. Machine Learning Techniques

To provide a deeper understanding of the methodologies employed in these studies, we will present a detailed description of each document and the specific machine-learning techniques they used. The following sections will outline the classifiers and optimization methods applied in each study, highlighting the diversity of approaches and the innovation in model development within the field of credit risk assessment.

- Ref. [15] applied Recursive Feature Elimination with Random Forest (RFE-RF) for feature selection, utilizing logistic regression, random forest, and SVM to improve model performance.
- Ref. [19] employed decision tree and logistic regression algorithms for evaluating credit status on P2P lending platforms, using the CfsSubsetEval evaluation strategy and BestFirst search strategy for feature selection.
- Ref. [25] utilized the Firefly Algorithm for feature selection in conjunction with KNN, Fuzzy KNN, Random Forest, Decision Tree, and SVM to enhance credit risk prediction accuracy.
- Ref. [14] combined Particle Swarm Optimization (PSO) with Structure Decision Tree Learning (SDTL) to improve financial credit risk assessment.
- Ref. [24] aimed to enhance interpretability in loan evaluation by extracting rules from a tree ensemble model using the NSGA-II algorithm.
- Ref. [20] employed *boosting* methods, including CatBoost, XGBoost, and LightGBM, to evaluate credit risk.
- Ref. [21] evaluated credit risk using decision tree and K-Nearest Neighbors (KNN) algorithms optimized through Bayesian optimization.
- Ref. [29] used heterogeneous ensemble learning to predict the default risk of national student loans, integrating algorithms like CatBoost, XGBoost, LightGBM, and Random Forest.
- Ref. [27] used the GBM Grid model to predict student payment behaviour at a private university, utilizing gradient boosting algorithms for classification.
- Ref. [31] employed various machine learning models, including SVM, logistic regression, random forest, Light Gradient Boosting (LGB), and eXtreme Gradient Boosting (XGB), for evaluating customer creditworthiness and monitoring credit repayments.
- Ref. [22] proposed the LGBBO-RuleMiner, a rule-based classification technique designed for predicting credit risk using a novel Biogeography Based Optimization (BBO) method.
- Ref. [12] combined Bolasso (Bootstrap-Lasso) with Random Forest, using the stability of Bolasso for feature selection and the classification power of Random Forest, along with SVM, Naïve Bayes, and K-Nearest Neighbors (K-NN).
- Ref. [9] employed a discrete Bayesian Network (BN) integrated with a latent variable for assessing credit risk by modelling the probability of loan default.
- Ref. [10] proposed a model that utilizes Gradient Boosting, a supervised learning method.
- Ref. [17] introduced the ABSMPNN model, which integrates Binarized Spiking Neural Networks (BSNN) with the Adaptive Marine Predators Algorithm (AMPA) for optimization, achieving high accuracy in credit risk evaluation with reduced computational time.
- Ref. [30] combined a Backpropagation (BP) neural network with a mutation genetic algorithm to improve the accuracy of credit risk assessment for commercial banks.
- Ref. [18] evaluated several machine learning models, including logistic regression, decision tree, random forest, SVM, and neural networks for credit risk assessment.
- Ref. [23] utilized logistic regression and neural network models within an automated ETL (Extraction, Transformation, Load) process to assess credit risk in compliance with Basel II standards, focusing on calculating Probability of Default (PD), Loss Given Default (LGD), and Exposure at Default (EAD).
- Ref. [28] integrated Wasserstein Generative Adversarial Networks (WGAN) with a hybrid feature selection approach combining Kernel Partial Least Square (KPLS) and Quantum Particle Swarm Optimization (QPSO), utilizing SVM, logistic regression, KNN, Adaboost, and Random Forest.
- Ref. [11] proposed the MIFCA model, which integrates multiple classifiers including decision tree, random forest, SVM, k-Nearest Neighbors (k-NN), BP Neural Network, and XGBoost to enhance the accuracy and robustness of credit risk assessment.
- Ref. [26] utilized deep neural networks to assess behavioural credit ratings, logistic regression, SVM, random forest, and XGBoost to meet Basel regulatory framework requirements.
- The Interpretable Selective Learning Framework proposed by [16] utilizes both logistic regression and neural networks. The framework enhances interpretability by selectively using the simpler logistic regression model where it is sufficient and the more complex neural network model where necessary.

A different approach compared to the others is presented by Zhao et al. [13]. While most other articles focus on classification tasks, this addresses data imputation. MGAIN is designed to handle missing data in credit risk assessment by utilizing a combination of Generative Adversarial Networks (GAN) for imputation.

**Figure 9:**
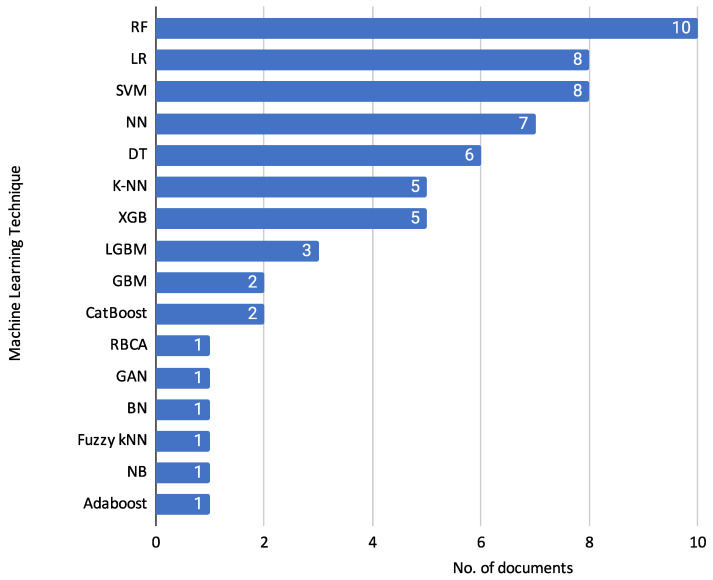
Frequency of Machine Learning Classifiers in Reviewed Documents.

Following the detailed descriptions of each document and their specific techniques, it is helpful to visualize the overall usage trends of the machine learning classifiers. Figure 9 summarises the techniques used and their reported frequency across the reviewed documents.

Figure 9 shows the frequency of various machine learning classifiers used in the reviewed documents. This diverse set of machine learning methods and algorithms highlights the varied approaches taken in the reviewed articles to tackle credit risk assessment.

Random Forest (RF) is the most frequently used classifier, appearing in 10 documents [11,12,15,18,24,25,26,28,29,31]. Logistic Regression (LR) is used in 8 documents [15,16,18,19,23,26,28,31], and Support Vector Machine (SVM) is also used in 8 documents [11,12,15,18,25,26,28,31]. Neural Networks (NN) are used in 7 documents [11,16,17,18,23,26,30]. Decision Trees (DT) are employed in 6 documents [11,14,18,19,21,25]. K-Nearest Neighbors (K-NN) [11,12,21,25,28] and eXtreme Gradient Boosting (XGB) [11,12,21,25,28] are each used in 5 documents. Light Gradient-Boosting Machine (LGBM) is used in 3 documents [20,29,31]. Gradient Boosting Machine (GBM) [10,27] and Categorical Boosting (CatBoost) [20,29] appear in 2 documents each. Finally, Rule-based Classification Algorithm (RBCA) [22], Generative Adversarial Networks (GAN) [28], Bayesian Network (BN) [9], Fuzzy k-Nearest Neighbors (Fuzzy kNN) [25], Naïve Bayes (NB) [12], and Adaptive Boosting (Adaboost) [28] are each used in 1 document.

#### 3.2.2. Credit Risk Assessment in Practice

Credit risk assessment and Machine Learning techniques are applied in various financial contexts, each with unique challenges and requirements. This section explores the application of these methodologies in traditional banking versus peer-to-peer (P2P) lending, highlighting their respective advantages and limitations. Exceptional cases such as student loan default are also examined to illustrate the specific methodologies used to predict and manage these unique risks.

This section explores research studies related to credit risk assessment within the financial services industry, specifically focusing on the banking sector.

- Ref. [17] Used in the banking sector to improve the accuracy and efficiency of identifying customer credit quality.
- Ref. [23] Focuses on automated credit risk assessment to enhance operational efficiency and compliance with regulatory standards in the financial sector.
- Ref. [15] Aims to optimize predictive accuracy and stability of credit scoring models.
- Ref. [12] Enhances the stability and accuracy of predictions regarding loan defaults.
- Ref. [13] Improves the accuracy and efficiency of credit risk assessment models by effectively handling missing data.
- Ref. [11] Enhances the accuracy and efficiency of personal credit risk predictions in the banking sector.
- Ref. [10] Integrates credit history, repayment behaviour, and social network data to improve creditworthiness assessment.
- Ref. [16] Aims to improve the interpretability and accuracy of credit risk predictions.
- Ref. [25] Focused on improving the accuracy and interpretability of credit card risk prediction.
- Ref. [14] Enhances prediction models for credit risk management in digital banking.
- Ref. [31] Designed to enhance transparency, fairness, and effectiveness of credit scoring systems in the financial domain.
- Ref. [26] Predicts the future performance of credit portfolios, focusing on behavioural patterns indicating a risk of default.
- Ref. [18] Uses data from the Central Bank Credit Registry to enhance predictive models for credit risk assessment.
- Ref. [9] Models payment default of loan subscribers using a Bayesian network with a latent variable.
- Ref. [24] Loan Evaluation with Tree Ensemble Models: Improves the interpretability of loan evaluation models while maintaining predictive performance.
- Ref. [30] Focuses on the credit risk assessment of commercial banks to improve decision-making processes.
- Ref. [28] focuses on improving credit risk assessment models by addressing challenges related to high-dimensional data and small sample sizes in emerging financial sectors.
- Ref. [20] Uses boosting methods for credit risk assessment, exploring the effectiveness of these methods on high-dimensional, weakly correlated, and sparse datasets.
- Ref. [22] Applied in financial institutions to improve decision-making processes regarding loan approvals.

Following examining credit risk assessment in traditional banking, this section investigates research on applying these techniques within the peer-to-peer lending sector.

- Ref. [19] Risk Evaluation and Management: Assesses credit risk on P2P lending platforms to improve risk evaluation and management.
- Ref. [21] Borrower Classification: Classifies borrowers to identify potential defaulters in P2P lending, enhancing the reliability of credit assessments.

Finally, this section examines research on credit risk assessment within the context of educational institutions.

- Ref. [29] Predicts the default risk on national student loans, providing insights for managing and mitigating risks.
- Ref. [27] Develops a classification model to predict student payment behaviour, which is crucial for financial planning and risk management in educational institutions.

### 3.3. Feature Selection Methods

Feature selection is a relevant step in building effective machine-learning models. It involves selecting the most relevant features from the dataset to improve model performance and interpretability. Following the extensive related literature [33,34,35,36,37,38,39,40,41,42,43,44], the solution methods for feature selection problem can be broadly categorized into three approaches: filter methods, wrapper methods, and embedded methods.

- **Filter Methods:** Filter methods evaluate the relevance of features by examining their statistical properties concerning the target variable. These methods are independent of any machine learning algorithm. Common filter methods include, Mutual Information: Measures the mutual dependence between features and the target variable; Chi-Squared Test: Assesses the association between categorical features and the target variable; Correlation Coefficient: Examines the linear relationship between numerical features and the target variable.
- **Wrapper Methods:** Wrapper methods evaluate feature subsets based on their performance with a specific machine learning algorithm. These methods involve training and evaluating a model for each subset of features. Common wrapper methods include, Recursive Feature Elimination (RFE): Iteratively removes the minor essential features based on model performance until the optimal feature subset is obtained; Forward Selection: Starts with an empty feature set and adds features one by one based on their contribution to model performance; Backward Elimination: Starts with all features and removes them one by one based on their lack of contribution to model performance.
- **Embedded Methods:** Embedded methods perform feature selection during the model training process. These methods are specific to particular algorithms and integrate feature selection as part of the model building. Standard embedded methods include Least Absolute Shrinkage and Selection Operator (LASSO): A linear model that performs L1 regularization, which can shrink some coefficients to zero, effectively selecting a subset of features; Tree-based Methods: Decision trees and ensemble methods like Random Forest and Gradient Boosting inherently perform feature selection by selecting essential features during the tree-building process; Elastic Net: Combines L1 and L2 regularization to choose a subset of features while maintaining some of their effects.

Filter methods, which evaluate the relevance of features based on statistical properties, are also widely used. Ref. [19] employs the CfsSubsetEval evaluation and BestFirst search strategies. Ref. [11] uses Pearson correlation analysis, and [10] applies KS and AUC for univariate analysis, followed by a correlation-based method to remove highly correlated features. Other examples include [31], which removes features affected by collinearity, and [18], which uses information value and correlation analysis.

Wrapper methods, which evaluate feature subsets based on their performance with a specific machine learning algorithm, are observed in [25] using the Firefly Algorithm and [14] using Particle Swarm Optimization (PSO). Additionally, ref. [27] employs Boruta for feature selection.

Several articles implement embedded feature selection methods, integrating feature selection within the model training process. Ref. [23] utilizes logistic regression and neural network models for embedded feature selection. Similarly, ref. [15] employs Recursive Feature Elimination (RFE) with Random Forest, and [12] uses Bolasso, which integrates feature selection within the learning process. Another approach is seen in [24], which uses Lasso, Ridge, ElasticNet, Feature Importance, and Chi-square for embedded feature selection.

A hybrid approach is seen in [28], which combines Kernel Partial Least Square (KPLS)-based filter and Quantum Particle Swarm Optimization (QPSO)-based wrapper for feature selection.

Some articles did not focus on feature selection methods. For instance, Ref. [13] concentrates on data imputation rather than feature selection.

These diverse methods highlight the importance of various feature selection techniques in improving model performance and interpretability in credit risk assessment and other applications.

### 3.4. Optimization Techniques (Metaheuristics)

Optimization techniques, particularly metaheuristics, are general-purpose algorithms that, with minor adaptations, can solve various optimization problems. They are characterized by finding high-quality solutions in reasonable times by balancing exploration and exploitation of the search space [45].

Currently, these optimization techniques are essential for fine-tuning machine learning models. These methods find the best model parameters that minimize or maximize a specific objective function. Common metaheuristic optimization techniques include Genetic Algorithms (GA), Particle Swarm Optimization (PSO), and Simulated Annealing (SA). In the reviewed articles, the following metaheuristics were observed:

- **Biogeography Based Optimization (BBO):** Biogeography Based Optimization (BBO) is inspired by the science of biogeography, which studies the distribution of species across different habitats over time [46]. In BBO, each potential solution to an optimization problem is considered a habitat with a habitat suitability index (HSI) representing its fitness. Habitats with high HSI share their characteristics with habitats with lower HSI, analogous to species migration in natural ecosystems. This exchange of features helps explore the search space and find optimal solutions.
- **Adaptive Marine Predators Algorithm (AMPA):** The Adaptive Marine Predators Algorithm (AMPA) mimics the adaptive foraging behaviour of marine predators [47]. This algorithm adapts the strategies of pursuit, encircling, and attacking prey based on the dynamic environment of the prey-predator interaction, enhancing its exploration and exploitation capabilities.
- **Variable Color Harmony Algorithm (VCHA):** The Variable Color Harmony Algorithm (VCHA) is inspired by the improvisation process of musicians when harmonizing different colours [48]. This algorithm adjusts its parameters dynamically to balance exploring new solutions and exploiting known reasonable solutions, optimizing complex functions effectively.
- **Quantum Particle Swarm Optimization (QPSO):** Quantum Particle Swarm Optimization (QPSO) is an advanced version of Particle Swarm Optimization (PSO) that incorporates principles of quantum mechanics [49]. In QPSO, particles have quantum behaviour, allowing them to explore the search space more effectively. The position of each particle is updated based on a probability distribution rather than a deterministic rule, which helps avoid local optima and find global solutions.
- **Firefly Algorithm (FFA):** The Firefly Algorithm (FFA) is inspired by the flashing behaviour of fireflies [50]. In this algorithm, the brightness of each firefly is associated with its fitness, and fireflies are attracted to brighter ones. The attractiveness decreases with distance, leading fireflies towards more glowing and optimal solutions. This behaviour enables effective exploration and exploitation of the search space.
- **Particle Swarm Optimization (PSO):** Particle Swarm Optimization (PSO) is a population-based optimization technique inspired by the social behaviour of birds flocking or fish schooling [51]. In PSO, each particle represents a potential solution and adjusts its position in the search space based on its own experience and the experience of neighbouring particles. The particles move towards better solutions over iterations, balancing exploration and exploitation to find the global optimum.
- **Non-dominated Sorting Genetic Algorithm II (NSGA-II):** NSGA-II is an advanced evolutionary algorithm specifically designed for solving multi-objective optimization problems [52]. It uses a fast, non-dominated sorting approach to classify solutions into different fronts based on Pareto dominance. Additionally, NSGA-II employs a crowding distance mechanism to ensure diversity among the solutions and a binary tournament selection based on the rank and crowding distance. This algorithm effectively balances convergence towards the Pareto front and diversity among the solutions, making it widely used in various optimization tasks where multiple conflicting objectives must be optimized simultaneously.
- **Genetic Algorithm (GA):** Genetic Algorithm (GA) is a popular metaheuristic inspired by the principles of natural selection and genetics [53]. It works by evolving a population of potential solutions over successive generations. Each individual in the population represents a candidate solution encoded as a chromosome. The algorithm uses selection, crossover (recombination), and mutation operators to generate new offspring. Selection chooses the fittest individuals to reproduce, crossover combines parts of two parents to create offspring, and mutation introduces random changes to maintain genetic diversity. GAs are highly effective for solving complex optimization problems because they can explore an ample search space and avoid local optima.

The LGBBO-RuleMiner proposed by [22] is a rule-based classification technique designed to predict credit risk using a novel Biogeography Based Optimization (BBO) method. This algorithm discovers an optimal rule set with high predictive accuracy from datasets containing both categorical and continuous attributes. BBO employs evolutionary operators like migration and mutation to generate and refine rules iteratively, ensuring a balance between exploration and exploitation of the search space. Ref. [17] introduced the ABSMPNN model for accurately identifying customer credit quality in the banking sector. It integrates Binarized Spiking Neural Networks (BSNN) with the Adaptive Marine Predators Algorithm (AMPA) for optimization. The model processes information with spiking neurons, optimized by AMPA, to maximize accuracy and minimize loss. Additionally, the Variable Color Harmony Algorithm (VCHA) enhances feature selection.

In the work by [28], a hybrid method integrates Wasserstein Generative Adversarial Networks (WGAN) for data augmentation with Kernel Partial Least Square (KPLS)-based filter and Quantum Particle Swarm Optimization (QPSO)-based wrapper for feature selection. This approach generates virtual samples to address data scarcity, ranks feature importance, and optimizes the feature subset to improve model performance. Ref. [25] utilized the Firefly Algorithm (FFA) for feature selection in conjunction with several classification techniques such as KNN, Fuzzy KNN, Random Forest, Decision Tree, and SVM. The Firefly Algorithm optimizes the subset of features to improve the performance of the classifiers, demonstrating its effectiveness in handling unbalanced data through the use of SMOTE for data balancing.

The model proposed by [14] combines the Particle Swarm Optimization (PSO) algorithm with Structure Decision Tree Learning (SDTL) to assess financial credit risk. The PSO algorithm optimizes the feature selection process, while SDTL provides robust classification capabilities, enhancing the accuracy and reliability of credit risk predictions.

In [24], a method aimed to enhance interpretability in loan evaluation by extracting rules from a tree ensemble model in two stages. The first stage involves local rule extraction, and the second stage optimizes the entire rule set using the NSGA-II algorithm, balancing predictive performance and interpretability.

Lastly, Ref. [30] combined a Backpropagation (BP) neural network with a mutation genetic algorithm to enhance the accuracy of credit risk assessment for commercial banks. The mutation genetic algorithm optimizes the network parameters, addressing issues like local minima and improving convergence speed.

An important aspect when combining different algorithms is their computational impact. Considering the large computational and energy costs, it is of great interest to determine how long an algorithm takes and how complex it is for solving problems. Along these lines, it can be seen that articles [17,28] demonstrate the efficiency of their algorithms through the computational time required for the experiments performed. It is striking that only two articles that use metaheuristic algorithms demonstrate their computational efficiency.

### 3.5. Datasets and Variables

Datasets are the backbone of credit risk assessment models, providing the necessary data for training and validation. This section provides an overview of commonly used public datasets, such as those from the UCI Repository and Kaggle, and custom/proprietary datasets from credit registries and commercial banks with the purpose of evaluate individual credit risk. It also discusses the challenges associated with data scarcity and imputation, which are critical for maintaining model accuracy and reliability.

**Figure 10:**
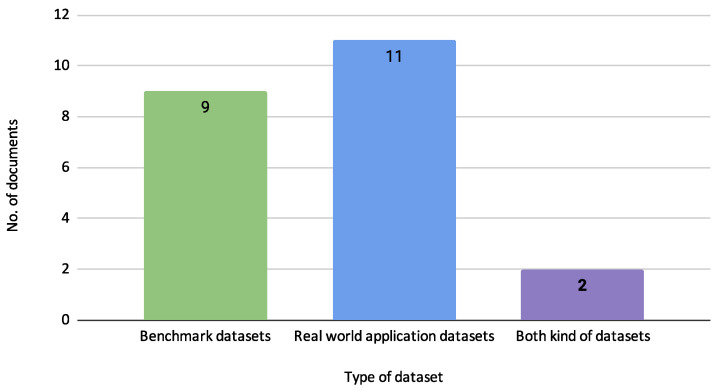
Usage of Benchmark vs. Real-World Application Datasets.

Figure 10 illustrates the usage distribution of benchmark datasets, real-world application datasets, and those that utilize both types.

As shown in Figure 10, the analysis includes nine studies that use benchmark datasets, 11 that use real-world application datasets, and two that utilize both types. This distribution highlights a balanced focus on theoretical benchmarking and practical application, ensuring the models are robust and applicable to real-world scenarios. The benchmark datasets provide a controlled environment for initial testing. In contrast, the real-world datasets offer insights into the practical performance and adaptability of the credit risk models in diverse financial contexts.

#### 3.5.1. Analysis of Commonly Used Datasets

Analyzing benchmark datasets is crucial for evaluating the performance and generalizability of credit risk models. These datasets provide a controlled environment for testing various methodologies, allowing for consistent comparison and validation across different studies.

**Figure 11:**
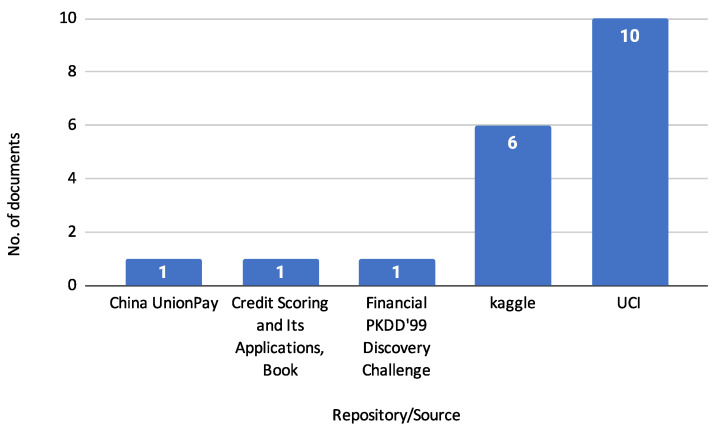
Sources of Benchmark Datasets.

Figure 11 illustrates this study’s sources of benchmark datasets. This figure shows that most benchmark datasets are cited from the UCI repository, appearing in 10 reviewed documents. Kaggle is the next most referenced source with six documents, followed by China UnionPay, the book *Credit Scoring and Its Applications* [1], and the Financial PKDD’99 Discovery Challenge, each cited in 1 document. This distribution highlights the prominence of UCI and Kaggle as key repositories for benchmark datasets in credit risk assessment. Table 5 describes the specific datasets from these sources.

Table 5 lists various public datasets used for credit scoring and risk analysis, detailing their sources, the number of instances, features, and labels. Notable entries include the Statlog datasets from the UCI repository, the Bank Loan Status dataset from Kaggle, and the Default of Credit Card Clients dataset, showcasing various sample sizes and feature counts suitable for benchmarking credit risk models.

- **Thomas dataset**: The Thomas dataset [1] includes various attributes commonly used in credit scoring models. This dataset is used to analyze credit risk and develop credit scoring models. Ref. [25] utilized this dataset to explore advanced credit scoring techniques and assess model performance under various conditions.
- **Statlog (German Credit Data)**: The Statlog (German Credit Data) [54] dataset is used to classify loan applicants as either good or bad credit risks based on various personal and financial attributes. This dataset has been extensively used in multiple studies, including [12,15,22,24,25], to test and validate different credit scoring models and machine learning algorithms.
- **Statlog (Australian Credit Approval)**: The Statlog (Australian Credit Approval) dataset [55] is used for credit scoring, facilitating quick decision-making and reducing risks associated with loan collections. It has been employed in various research works, such as [22,24], to evaluate the effectiveness of credit approval models.
- **South German Credit Dataset**: The South German Credit Dataset [56] contains similar financial and personal attributes as the German Credit Data used for creditworthiness assessment. This dataset is utilized in [25] to examine the robustness of credit scoring methodologies.
- **Loan Default Prediction Dataset**: Used for predicting loan defaults, the Loan Default Prediction dataset [57] serves as a binary classification problem. It is referenced in [28] for developing and testing predictive models for loan default.
- **Kaggle’s Bank Loan Status Dataset**: This dataset [58] contains data related to bank loans and is used to classify loan status as good or bad, aiding in decision-making for loan approval. Ref. [12] explored this dataset to improve loan approval processes.
- **Kaggle Home Credit Default Risk**: The Kaggle Home Credit Default Risk dataset [59] aims to predict a client’s ability to repay loans based on transaction and credit information. Ref. [20] utilized this dataset to enhance credit risk assessment models.
- **Give Me Some Credit Dataset**: Designed to predict default payments, the Give Me Some Credit dataset [60] includes comprehensive variables that reflect the financial history of the borrower. Ref. [16] employed this dataset to develop interpretable credit risk models.
- **Default of Credit Card Clients**: Collected from credit card holders of a bank in October 2015, this dataset [61] focuses on default payments. Refs. [16,21] used this dataset, often referred to as the Taiwan credit dataset, for credit scoring and risk analysis.
- **Czech Financial Dataset**: The Czech Financial Dataset [62] consists of various credit card application decisions, encoded and anonymized. This dataset was used in [31] to study credit risk and develop predictive models.
- **Credit Risk Dataset**: This dataset [63] includes comprehensive economic records of financial businesses and their related evaluation outcomes. Ref. [17] utilized this dataset for predicting credit risk and improving credit evaluation methodologies.
- **Credit Card Econometrics**: The Credit Card Econometrics dataset [64] focuses on credit card usage and repayment behaviours for risk assessment. Ref. [25] explored this dataset to enhance credit scoring techniques.
- **China UnionPay Credit Dataset**: Obtained from a data competition created by China UnionPay [65], this dataset analyses credit risks, focusing on both good and bad credit observations. [28] utilized this dataset to improve credit risk prediction models.

Table 6 lists various datasets used in real-world applications, detailing their sources, number of instances, features, and labels. These datasets are relevant for developing and validating credit scoring, risk assessment, and financial analysis models. This table presents various datasets, each with its unique application in real-world scenarios.

The Lending Club Loan Data and Lending Club Dataset are sourced from Lending Club and contain numerous features that can be used to analyze loan statuses. The Credit Risk Assessment Data from an anonymous local bank in China and the Commercial Bank Credit Records dataset provide insight into credit risk and customer profiles. Additionally, datasets like the Croatian Bank Credit Risk Dataset and the North Macedonia Credit Registry Data offer extensive records for detailed financial analysis. The diversity and richness of these datasets make them invaluable for financial modelling and risk assessment research.

- **Lending Club Loan Data**—This dataset contains consumer loans from 2007 to 2018 from the Lending Club. It was used in [23].
- **Lending Club Dataset**—Includes records from loans issued by Lending Club between 2007 and 2011. Referenced in [12].
- **Credit Risk Assessment Data**—Private data of credit risk assessment provided by a local bank in China. Utilized in [13].
- **LendingClub (LC)**—The dataset includes comprehensive loan data from Lending Club from 2018. It is used to evaluate the credit risk of borrowers on the P2P lending platform, as seen in [19].
- **Commercial Bank Credit Records**—Contains personal loan application records used for credit risk assessment. Referenced in [11].
- **Business Credit Score Dataset**—Comprises financial and social interaction data of companies from their first loan and observed over 12 months. Used in [10].
- **Personal Credit Score**—Comprises financial and social interaction data of individuals from their first loan and observed over 12 months. Referenced in [10].
- **General Data Protection Regulation (GDPR)**—Reflects monthly credit statuses for certain clients until the planned completion of their loans. Referenced in [14].
- **Advanced Analytics of Credit Registry Dataset**—This dataset includes properties, dependencies, trends, and advanced analytics features for in-depth data analysis. Referenced in [14].
- **WIND Dataset**—Covers personal credit data and is used for PSO-SDTL model analysis. Referenced in [14].
- **Croatian Bank Credit Risk Dataset 2009–2013**—The dataset tracks loan information annually and predicts default events within a year following each annual snapshot. Utilized in [26].
- **Croatian Bank Credit Risk Dataset 2004–2018**—Similar to the 2009-2013 dataset, it tracks annual loan information to predict default events. Referenced in [26].
- **North Macedonia Credit Registry Data**—Central to all credit activities in the country, capturing monthly credit and credit card status for various clients, aggregated from all commercial banks. Referenced in [18].
- **Tunisian Bank Loan Data**—Describes loan contracts granted by several Tunisian banks from 1990-2012. Referenced in [9].
- **Lending Club (LC) Dataset 2017–2018**—Includes records of loans to evaluate the performance of machine learning models in predicting loan defaults. Utilized in [24].
- **Bank Credit Risk Data**—Contains financial and non-financial information used to assess the credit risk of loan enterprises. Referenced in [30].
- **National Student Loans Dataset**—Data collected on students who had applied for national student loans, including personal honours, GPA by semester, and loan information, tracked until May 2022. Referenced in [29].
- **Student Payment Behavior Dataset**—Students have been considered participants for predicting payment behaviour in students of a private university in Peru in 2022. Utilized in [27].

#### 3.5.2. Summary of Variables Commonly Used

Following the dataset discussion, this subsection focuses on the variables frequently utilized in credit risk assessment models. The variables are categorized into several key groups: demographic information, financial status, loan specifics, credit history, employment details, educational background, and loan default behaviour. This organization provides a clear overview of the diverse data points for building accurate and reliable credit risk models. It is important to note that not all studies in the review declare all variables, and the relevance of each variable in the models is not always specified. Therefore, we work with the information that is declared and available. By systematically categorizing these variables, we highlight the significant predictors of borrower creditworthiness and offer insights into the multifaceted nature of credit risk assessment.

##### **Demographic Information** 

Demographic variables include characteristics such as age, gender, and marital status. These variables provide essential context about the personal background of the borrower. Common variables in this category are:

- Age: The age of the borrower (e.g., Lending Club dataset, Statlog (German Credit Data)).
- Gender: The gender of the borrower (e.g., Default of Credit Card Clients).
- Marital status: Whether the borrower is single, married, or divorced (e.g., Default of Credit Card Clients).
- Personal status and sex: Combined information on sex and marital status (e.g., Statlog (German Credit Data)).

##### **Financial Information** 

Financial status variables such as income and savings capture the borrower’s economic situation. These variables are critical in assessing the borrower’s loan repayment ability. Common variables include:

- Annual Income: The annual income reported by the borrower (e.g., Lending Club dataset, Kaggle’s Bank Loan Status dataset).
- Monthly debt: The amount of debt the borrower is paying monthly (e.g., Kaggle’s Bank Loan Status dataset).
- Savings account/bonds: The savings the borrower holds (e.g., Statlog (German Credit Data)).

##### **Loan Information** 

Variables related to the loan specifics provide details about the loan itself, such as the loan amount, term, and purpose. These variables help in understanding the nature of the loan. Examples include:

- Loan amount: The total loan amount applied for (e.g., Lending Club dataset, Statlog (German Credit Data)).
- Term: The loan duration in months (e.g., Lending Club dataset, Kaggle’s Bank Loan Status dataset).
- Purpose: The reason for which the loan is taken (e.g., Lending Club dataset, Statlog (German Credit Data)).

##### **Credit History** 

Credit history variables document the borrower’s past credit behaviour, which is crucial for predicting future behaviour. Key variables are:

- Credit history: The history of compliance with previous or concurrent credit contracts (e.g., Statlog (German Credit Data), Default of Credit Card Clients).
- Number of open accounts: The number of open credit lines in the borrower’s credit file (e.g., Lending Club dataset).
- Delinquencies: The number of delinquent accounts in the borrower’s credit file (e.g., Lending Club dataset, Default of Credit Card Clients).

##### **Employment Information** 

Employment-related variables provide insight into the borrower’s job stability and income source. Important variables include:

- Employment length: The duration of the borrower’s current employment (e.g., Lending Club dataset, Default of Credit Card Clients).
- Job: The type of job the borrower holds (e.g., Statlog (German Credit Data), Tunisian Bank Loan Data).

##### **Educational Information** 

Educational background variables offer information about the borrower’s education level, which can indicate income potential. Common variables are:

- Education level: The highest level of education the borrower completes (e.g., Default of Credit Card Clients).
- GPA: The borrower’s grade point average (e.g., loan default risk for college students dataset).

##### **Loan Default Behavior** 

Loan default behaviour variables indicate whether the borrower has defaulted on loans. This category is critical for risk assessment. Typical variables include:

- Default: Whether the borrower has defaulted on a loan (e.g., Default of Credit Card Clients, Tunisian Bank Loan Data).
- Loan status: The current status of the loan (e.g., Lending Club dataset, Kaggle’s Bank Loan Status dataset).

#### 3.5.3. Relevant Variables and Their Impact on Prediction

The datasets utilized in credit risk assessment provide a wealth of information and highlight certain variables crucial for accurate prediction. This section discusses the importance of specific variables as reported in the articles reviewed. Based on the findings in the literature, the importance is ranked from 1 (most important) to n (less important).

- **Statlog (Australian Credit Approval) and Statlog (German Credit Data)**: While the reviewed articles did not determine the specific importance of variables for these datasets, they remain pivotal for benchmarking credit scoring models due to their comprehensive feature sets.
- **Credit Risk Dataset**: The dataset includes several personal and loan-specific variables, though the articles did not specifically rank their importance. These variables are a person’s age, yearly income, homeownership, employment length (in years), loan purpose, loan rating, amount of loan, interest rate, loan status (0 for reject, 1 for approve), percentage revenue, ancient default, and credit history length. These features collectively contribute to the comprehensive credit risk assessment, providing critical insights into the borrower’s financial stability and creditworthiness.
- **Lending Club Loan Data**: This dataset includes various financial and demographic variables, but the reviewed articles did not detail their specific predictive impact.
- **Give Me Some Credit Dataset**: The following variables were identified as having significant predictive impact: Number of times the borrower has been 90 days or more past due (importance: 1); Number of times borrower has been 60–89 days past due but no worse in the last two years (importance: 2); Number of times borrower has been 30–59 days past due but no worse in the previous two years (importance: 3); Number of open loans (importance: 4) and Monthly debt payments, alimony, and living costs divided by monthly gross income (importance: 5).
- **Loan Default Risk for College Students**: In this dataset, the following variables were reported to have significant predictive importance: Total amount of scholarship (importance: 1); 5th semester GPA (importance: 2); Score obtained in the college entrance examination (importance: 3); Examinee category (importance: 4) and 4th semester GPA (importance: 5).
- **North Macedonia Credit Registry Data**: This dataset is notable for its detailed financial records, with the following variables identified as particularly impactful: Days delayed (importance: 1); Successfully paid loans (importance: 2); Loan duration in years (importance: 3); Actual year loan (importance: 4) and Interest rate (importance: 5).
- **Tunisian Bank Loan Data**: For this dataset, the following variables were highlighted for their impact: Amount of credit (importance: 1) and related to Credit type (importance: 2); Credit duration (importance: 3) and related to Job of households (importance: 4). The relatedness of these variables indicates that while each variable has its importance score, their predictive power is also influenced by their connections to other variables. These connections highlight that the impact of one variable can propagate through its links with different variables, amplifying or modifying its effect on the prediction of loan defaults. The relationship is illustrated by a network graph by [9], which shows how the variables interact and contribute collectively to the prediction model.
- **Thomas Dataset**: The Thomas dataset, used for advanced credit scoring techniques, includes several important variables, although specific rankings were not provided.

In summary, variables related to payment history, income, credit duration, and specific loan details are consistently identified as significant predictors in credit risk assessment models. Understanding the relative importance of these variables can help refine predictive models and improve the accuracy of credit risk assessments.

#### 3.5.4. Challenges Associated with Data Scarcity and Imputation

Data scarcity and the presence of missing values pose significant challenges in credit risk assessment. This subsection examines methods for handling incomplete data and addressing class imbalance, both of which are crucial for ensuring robust and reliable model performance.

Zhao et al. [13] tackle the issue of missing data through a novel imputation technique called Multiple Generative Adversarial Imputation Networks (MGAIN). In credit risk datasets, missing values often arise from customer omissions, data collection errors, or even fraud, severely undermining model reliability. Traditional imputation techniques, such as mean, mode, or regression imputation, while simple, frequently introduce bias by distorting the original data distribution.

More advanced machine learning techniques—such as K-nearest neighbours (KNN) and support vector regression (SVR)—improve on statistical methods but still struggle with scalability and complexity in high-dimensional datasets. Deep learning-based approaches, including back-propagation (BP) neural networks and generative adversarial networks (GANs), are increasingly used for their ability to model non-linear relationships among attributes.

MGAIN builds upon Generative Adversarial Imputation Networks (GAIN) introduced by Yoon et al. [66], which learn the distribution of observed data to generate realistic imputations. While GAIN is effective, it requires large training datasets and involves complex network structures. MGAIN addresses these limitations by partitioning attributes into subsets to increase the usable data, imputing missing values for each subset with GAIN, and synthesizing results using a weighted average. This approach simplifies the model, reduces data requirements, and yields more stable imputations. Empirical validation demonstrates that MGAIN outperforms both traditional GAIN and other imputation techniques on real-world credit risk datasets.

Another major challenge in credit scoring is class imbalance. Studies such as Hassani et al. [25] and Wang et al. [11] employ the Synthetic Minority Oversampling Technique (SMOTE) to mitigate this issue. In Hassani et al. [25], SMOTE is applied before feature selection with a hybrid firefly algorithm and classification using models such as KNN, FKNN, Random Forest, Decision Tree, and SVM. Similarly, Wang et al. [11] note that 96% of their real-world dataset belongs to the majority class and use SMOTE to synthesize minority-class samples. Both studies apply SMOTE to balance the datasets while avoiding simple duplication, which can increase overfitting risk.

Despite these efforts, neither study explicitly analyzes the impact of SMOTE on minority-class metrics such as recall or F1-score. For instance, although Wang et al. [11] report F1-scores for each class, they do not provide pre-SMOTE comparisons to quantify improvement. This omission reflects a broader pattern in the literature: the effects of resampling techniques are often assumed but rarely validated through class-disaggregated metrics. Consequently, claims about SMOTE’s benefits for minority class performance remain speculative in many cases.

Beyond data-related challenges, complex learning algorithms themselves have inherent limitations. High-capacity models such as neural networks and GANs offer strong predictive power but are prone to overfitting, especially when training data is sparse or noisy. Similarly, while SMOTE can alleviate class imbalance, it may also amplify noise or lead to overfitted decision boundaries if used without caution. These issues highlight the importance of robust validation procedures—such as cross-validation, regularization, and careful hyperparameter tuning—to ensure that credit scoring models generalize well to unseen data.

#### 3.5.5. Relevance to Individual Credit Risk Assessment

The reviewed studies encompass a variety of datasets, both public and proprietary, yet all meet the inclusion criteria of addressing individual credit risk. Public benchmark datasets such as Statlog (German Credit Data), Statlog (Australian Credit Approval), the Default of Credit Card Clients, and Kaggle’s Loan Default Prediction datasets are explicitly designed to evaluate the creditworthiness of individual borrowers. These datasets include personal, financial, and behavioral variables that are essential for modeling individual risk, such as age, employment length, income, credit history, and loan repayment behavior.

Likewise, real-world datasets used in the reviewed articles—such as those from Lending Club, commercial banks in China and Latin America, and national credit registries—also focus on individual-level credit decisions, typically in the form of personal loans, credit scores, or repayment tracking. These datasets provide rich, granular data used to assess loan eligibility, predict defaults, and model borrower behavior in practical settings.

Although a direct performance comparison under unified conditions was not feasible due to differences in preprocessing, feature selection, and evaluation metrics across studies, our review identifies consistent variable patterns across datasets. Key predictive features like delinquency history, loan amount, and employment status frequently appear across models, reinforcing their relevance to individual credit risk modeling.

This alignment between dataset content and the objective of assessing individual credit risk provides a solid basis for inclusion in our systematic review and supports the interpretability and generalizability of findings.

#### 3.5.6. Limited Study on Dataset Used

An important aspect when working with predictive models is the quality of the dataset used. Analyzing the dataset used is essential to detect potential issues that could lead to errors in the learning task. In this sense, the works [11,14,16,19,20,22,24,25,27,28] only present a description of the dataset used, omitting any prior manipulation to perform the learning tasks.

It is striking that few studies analyze the dataset used, such as the [17] study, where the authors present data preprocessing and the feature selection process. In [23], the authors perform a dependence and independence analysis of the variables. In [15], the authors present the normalization procedure for the variables before submitting them to the classifier. In [12], the researchers present how they preprocessed the data and standardized the variables. In [13], they present data preprocessing, which led to data imputation. In [10], the authors present the target variable and the feature engineering used. They present the data preprocessing work in [21,29,31]. On the other hand, the works [9,26,30] only present an analysis of the variables that make up the dataset through graphs with the objective of showing the distributions of the data.

The most comprehensive work is that carried out in [18], where the researchers present a complete analysis of the dataset, explaining how they obtained the data, the preprocessing performed, data transformation, treatment of missing data and outliers, feature selection, feature engineering, and finally, variable correlation.

### 3.6. Evaluation Metrics

Evaluating the performance of different algorithms is essential for identifying the most effective approaches to credit risk assessment. This section introduces the critical evaluation metrics based on the work of [67], which presents a taxonomy for organizing these metrics. The taxonomy categorizes the metrics into four main groups: performance metrics, metaheuristics metrics, feature metrics, and statistical test metrics. Additionally, the discussion includes newly identified metrics within these categories.

**Figure 12:**
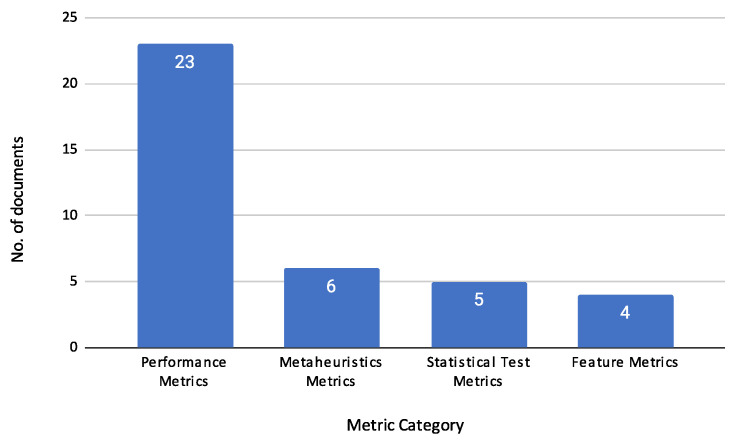
Distribution of Reported Metrics by Category.

Figure 12 shows the distribution of previously and newly defined metrics across the four categories described earlier. The Performance Metrics category has the highest reported metrics, with 23 documents. Metaheuristics Metrics follows with six documents reporting metrics related to metaheuristic algorithms. The Statistical Test Metrics and Feature Metrics categories are less common, with five and four documents respectively.

**Figure 13:**
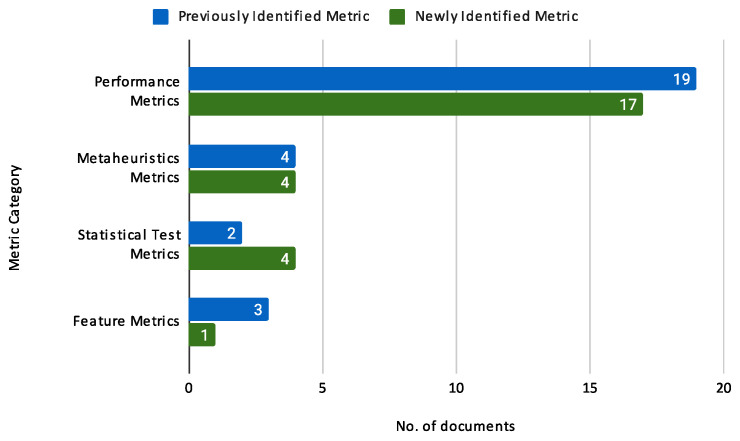
Comparison of previously identified metrics and newly identified metrics by Category.

Figure 13 presents the distribution of metrics across the four categories, distinguishing between those previously established and those newly identified in the reviewed literature. The results indicate a notable expansion of metric usage, particularly within the Performance Metrics category, where 17 documents introduced new metrics and 19 referred to previously recognized ones. In the Metaheuristics Metrics category, both new and existing metrics were reported in four documents each. The Statistical Test Metrics category shows a predominance of new metrics, with four documents reporting novel measures compared to two citing established ones. The Feature Metrics category includes one document that introduced a new metric and three that employed previously defined metrics. These findings underscore the necessity of revisiting and refining existing taxonomies to ensure they accurately represent the current methodological and evaluative practices observed in the literature.

Subsequently, we will detail the previously identified metrics, including the documents in which they appear and, where applicable, their mathematical formulations.

#### 3.6.1. Previously Identified Metrics

**Figure 14:**
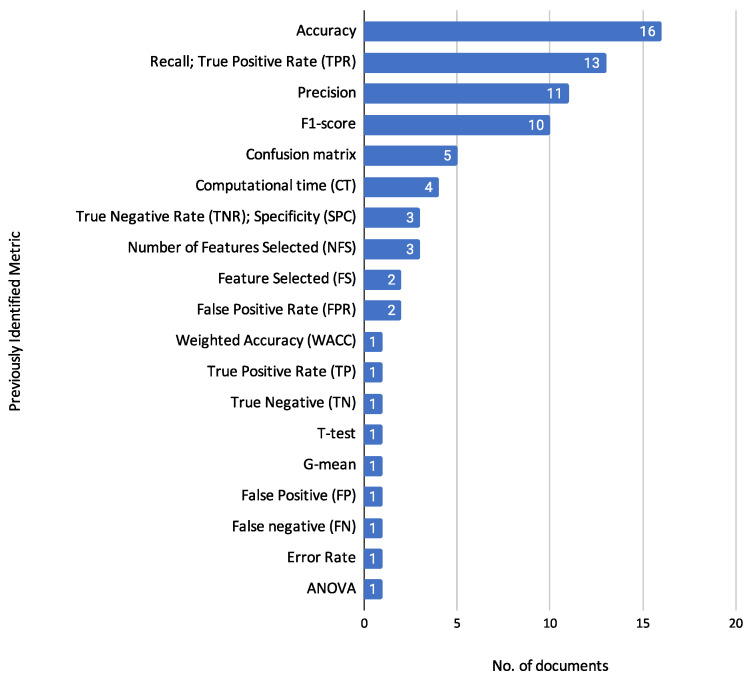
Trend of Previously Defined Metrics Distribution in Documents.

Concerning categorising previously identified metrics, Figure 14 illustrates the frequency of various reported metrics used in the reviewed documents. The Accuracy metric is the most frequently reported, appearing in 16 papers. This is followed closely by Recall (True Positive Rate, TPR), which is reported in 13 documents. Precision is mentioned in 11 papers, and the F1 score is reported in 10 documents. The Confusion Matrix appears in 5 papers, while Computational Time (CT) is reported in 4 documents.

Metrics such as True Negative Rate (TNR), also known as Specificity (SPC) and Number of Features Selected (NFS) are each reported in 3 documents. The Feature Selected (FS) metric and False Positive Rate (FPR) appears in 2 papers respectively.

Less frequently reported metrics, each appearing in 1 document, include Weighted Accuracy (WACC), True Positive Rate (TP), True Negative (TN), T-test, G-mean, False Positive (FP), False Negative (FN), Error Rate, and ANOVA. This distribution indicates a strong focus on accuracy and related performance metrics, underscoring their importance in the analyzed documents.

##### **Performance Metrics:** 

Performance metrics are critical for assessing the accuracy and effectiveness of classifiers used in credit risk assessment. These metrics help in understanding how well an algorithm can predict credit risk.

- **Confusion Matrix**: Used in studies [11,16,17,21,23], it provides a comprehensive view of the performance of an algorithm by displaying true positives, false positives, true negatives, and false negatives. The confusion matrix is a relevant tool for evaluating the performance of a classification model, providing a detailed breakdown of the model predictions compared to the actual outcomes. Table 7 shows a confusion matrix structure:
  Where:
  – TP (True Positives): The number of positive instances correctly classified as positive.
  – FN (False Negatives): The number of positive instances incorrectly classified as negative.
  – FP (False Positives): The number of negative instances incorrectly classified as positive.
  – TN (True Negatives): The number of negative instances correctly classified as negative.
  In [31], TP, TN, FP, and FN were used to compare the performance of different algorithms.
- **Accuracy**: Reported in multiple studies [9,11,12,14,15,17,18,19,20,21,22,23,25,29,30,31], it measures the proportion of correctly classified instances. Accuracy has the following formulation:
  (1) $$\mathrm{Accuracy}=\frac{\mathrm{TP}+\mathrm{TN}}{\mathrm{TP}+\mathrm{TN}+\mathrm{FP}+\mathrm{FN}}$$
  In [28], a variation called Weighted Accuracy (WACC) is presented, which is useful for imbalanced datasets. WACC is calculated based on the accuracy of different classes weighted by the class proportions. WACC is defined as:
  (2) $$\mathrm{WACC}=\sum_{i=1}^{N}w_{i}\cdot {\mathrm{Accuracy}}_{i}$$
  where $w_{i}$ is the weight of class *i* and ${\mathrm{Accuracy}}_{i}$ is the accuracy of class *i*.
- **Precision**: Found in studies [9,11,15,17,18,20,21,22,24,25,29], it indicates the number of true positive results divided by the number of all positive results. The precision has the following formulation:
  (3) $$\mathrm{Precision}=\frac{\mathrm{TP}}{\mathrm{TP}+\mathrm{FP}}$$
- **Recall - True Positive Rate (TPR) - Sensitivity**: Reported in studies [9,11,15,16,17,18,20,21,22,24,25,28,29], it measures the ability of a model to identify all relevant instances. Recall it has the following formulation
  (4) $$\mathrm{Recall}=\frac{\mathrm{TP}}{\mathrm{TP}+\mathrm{FN}}$$
- **F1-Score**: Included in studies [9,11,15,17,18,20,21,22,25,29], it is the harmonic mean of precision and recall. F1-score it has the following formulation.
  (5) $$F1\;\mathrm{Score}=2\cdot \frac{\mathrm{Precision}\cdot \mathrm{Recall}}{\mathrm{Precision}+\mathrm{Recall}}$$
- **False Positive Rate (FPR)**: Reported in studies [22,23], it measures the proportion of negatives incorrectly classified as positives. FPR has the following formulation.
  (6) $$\mathrm{FPR}=\frac{\mathrm{FP}}{\mathrm{FP}+\mathrm{TN}}$$
- **True Negative Rate (TNR) - Specificity**: Found in studies [17,21,28], it measures the proportion of actual negatives correctly identified. TNR has the following formulation.
  (7) $$\mathrm{TNR}=\frac{\mathrm{TN}}{\mathrm{TN}+\mathrm{FP}}$$
- **Error Rate**: Reported in [16], it measures the proportion of incorrectly classified observations. The error rate is calculated as:
  (8) ErrorRate=1−Accuracy
- **G-Mean**: Found in [28], it is the geometric mean of sensitivity and specificity. G-Mean is calculated as:
  (9) $$G-\mathrm{mean}=\sqrt{\mathrm{Type}\;I\;\mathrm{accuracy}\cdot \mathrm{Type}\;\mathrm{II}\;\mathrm{accuracy}}$$

##### **Metaheuristics Metrics:** 

Metaheuristics metrics evaluate the performance of optimization algorithms used in credit risk assessment.

- **Computational Time (CT)**: Reported in studies [12,15,17,28,30], CT measures the time taken by an algorithm or model to train, reach a solution, or make a prediction. This metric is crucial for evaluating the efficiency of an algorithm, especially in large-scale or real-time applications, where shorter computational times are often desired.

##### **Feature Metrics:** 

Corresponds to the number of features that comprise the best subset of features [67].

- **Number of Features Selected (NFS)**: Found in studies [12,25,28], Corresponds to the number of features that make up the best subset of feature.
  (10) NFS=|S|
  where S is the subset of selected features.
- **Feature selected (FS)**: Mentioned in [12,25], FS corresponds to the identification of the selected characteristics. This metric indicates which specific features are chosen from the original set for model building. The notation for FS can be represented as:
  (11) $$S=\{f_{1},f_{2},\ldots ,f_{k}\}$$
  where S is the subset of selected features and $f_{1},f_{2},\ldots ,f_{k}$ are the individual features in the subset.

##### **Statistical Test Metrics:** 

Statistical tests assess the significance of differences between models or features.

- ***t*****-test**: Reported in studies [13], it compares the means of two unrelated groups.
- **ANOVA (Analysis of Variance)**: Found in studies [17], it tests for significant differences between group means.

#### 3.6.2. Newly Identified Metrics

In the reviewed documents, we identified new metrics not previously considered in the categories defined by [67]. However, we believe these metrics can be integrated into the existing categories, expanding the current classification. These new metrics provide additional insights and tools for evaluating model performance, metaheuristics metrics, feature evaluation, and statistical tests, contributing to a more comprehensive assessment framework.

**Figure 15:**
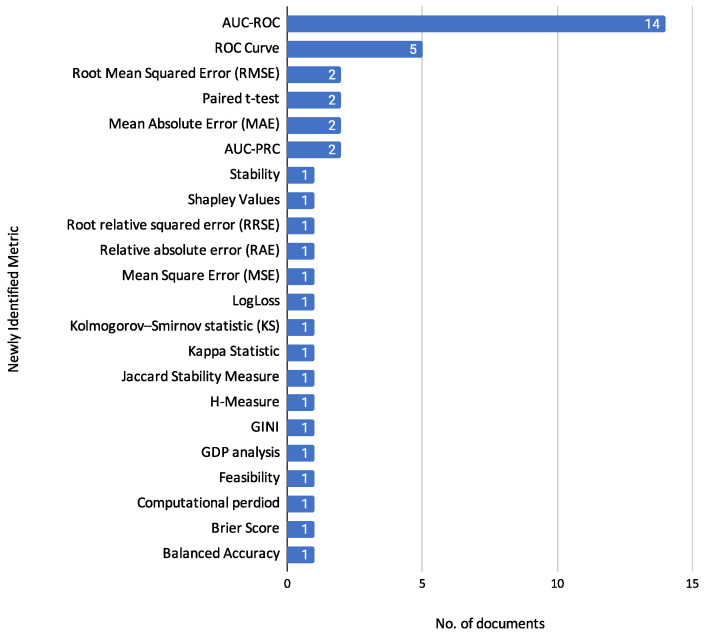
Trend of Newly Identified Metrics Distribution in Documents.

Figure 15 illustrates the distribution of newly identified metrics used in the reviewed documents. The AUC-ROC metric is the most frequently reported, appearing in 14 papers. The ROC Curve is reported in 5 documents. Metrics such as Root Mean Squared Error (RMSE), Paired *t*-test, Mean Absolute Error (MAE), and AUC-PRC are each reported in 2 papers. Less frequently reported metrics, each appearing in 1 document, include Stability, Shapley Values, Root Relative Squared Error (RRSE), Relative Absolute Error (RAE), Mean Square Error (MSE), Log Loss, Kolmogorov–Smirnov statistic (KS), Kappa Statistic, Jaccard Stability Measure, H-Measure, GINI, GDP analysis, Feasibility, Computational Period, Brier Score, and Balanced Accuracy. This distribution indicates the diverse range of metrics now being utilized, highlighting the evolving methodologies within the field.

Next, we will present these newly identified metrics, along with the documents in which they appear and, where applicable, their mathematical formulations.

##### **Performance Metrics:** 

We present the new metrics detected in the reviewed documents that can be added to the Performance Metrics category. These metrics enhance our ability to evaluate the effectiveness and accuracy of machine learning models across different applications. They include advanced measures for assessing predictive performance, error analysis, and class-specific outcomes.

- **Balanced Accuracy**: Found in [24]. Balanced accuracy is defined in [68] as the average of the true positive rate (sensitivity) and the true negative rate (specificity). It provides a more balanced performance measure by considering the correctly predicted positive and negative instances.
  (12) $$\mathrm{Balanced}\;\mathrm{Accuracy}=\frac{1}{2}\left(\frac{\mathrm{TP}}{\mathrm{TP}+\mathrm{FN}}+\frac{\mathrm{TN}}{\mathrm{TN}+\mathrm{FP}}\right)$$
- **Receiver Operating Characteristic Curve (ROC Curve)**: Used in [16,17,18,23,29]. The ROC curve is a graphical plot that illustrates the performance of a binary classifier system as its discrimination threshold is varied. As is illustrated in [69], the ROC curve is constructed by plotting pairs of False Positive Rate (FPR, defined in Equation (6)) and True Positive Rate (TPR, defined in Equation (4)) for all possible cut-off values *c*. Formally, it is represented as:
  (13) ROC(·)={FPR(c),TPR(c)∣c∈(−∞,+∞)}
- **Area Under the Receiver Operating Characteristic Curve (AUC-ROC)**: Seen in studies [10,12,16,17,20,21,22,23,24,26,27,28,29,31], it evaluates the ability of the model to discriminate between classes. A mathematical definition is provided by [69], where AUC is defined as:
  (14) $$\mathrm{AUC}=\int_{0}^{1}\mathrm{ROC}\left(t\right)\;dt$$
  where ROC(t) is the function describing the ROC curve, and *t* represents the threshold. The AUC value ranges from 0 to 1, with higher values indicating better model performance.
- **Area Under the Precision-Recall Curve (AUC-PRC)**: Reported in [22,27], it evaluates the trade-off between precision and recall for different threshold values. We present the following formulation for the AUC-PRC, calculated as the area under the precision-recall curve:
  (15) $$\mathrm{AUC}\text{-}\mathrm{PRC}=\int_{0}^{1}\mathrm{PRC}\left(t\right)\;dt$$
  where PRC(t) is the precision-recall curve as a function of the threshold *t*.
- **Kolmogorov–Smirnov Statistic (KS)**: Used in [10] to evaluate the performance of the model. It is a non-parametric test that quantifies the difference between the distribution of predictions for different classes [70]. The KS statistic is defined as:
  (16) $$\mathrm{KS}=\max|F_{\mathrm{positive}}\left(x\right)-F_{\mathrm{negative}}\left(x\right)|$$
  where $F_{\mathrm{positive}}\left(x\right)$ and $F_{\mathrm{negative}}\left(x\right)$ are the empirical cumulative distribution functions of the predicted scores for the positive and negative classes, respectively. A higher KS value indicates better model discrimination between the classes.
- **Kappa Statistic**: As reported in [22], the Kappa statistic measures the agreement between predicted and observed categorizations. It is calculated as:
  (17) $$\mathrm{Kappa}=\frac{P_{o}-P_{e}}{1-P_{e}}$$
  where $P_{o}$ is the observed agreement and $P_{e}$ is the expected agreement. These are calculated as $P_{o}=\frac{\mathrm{NRA}}{\mathrm{TNI}}$, where NRA is the Number of times both raters agree, and TNI is the Total number of instances. The expected agreement is $P_{e}=\sum_{i=1}^{k}\left(P_{i1}\cdot P_{i2}\right)$, where *k* is the number of categories, $P_{i1}$ is the proportion of instances assigned to the *i*-th category by the first rater, and $P_{i2}$ is the proportion assigned by the second rater.
- **GINI**: Found in [27], it measures the inequality among values of a frequency distribution. This metric evaluates the performance of credit risk assessment models in distinguishing between different credit risk levels. The GINI coefficient summarizes the dispersion or inequality commonly used in economic studies. A higher GINI coefficient indicates better classification ability of the model. The article did not provide a specific mathematical formulation.
- **GDP analysis**: Found in [14], it is used to evaluate the economic impact of the proposed credit risk assessment models. By analyzing GDP, the study assesses the effectiveness of these models in improving financial stability and economic performance. It is important to note that no mathematical description of the GDP analysis metric is provided in the article.
- **Mean Absolute Error (MAE)**: Mentioned in studies [13,22], it measures the average magnitude of errors in a set of predictions. MAE is calculated as:
  (18) $$\mathrm{MAE}=\frac{1}{n}\sum_{i=1}^{n}\left|{\hat{y}}_{i}-y_{i}\right|$$
  where ${\hat{y}}_{i}$ is the predicted value, $y_{i}$ is the actual value, and *n* is the total number of observations.
- **Mean Square Error (MSE)**: Mentioned in [14], MSE evaluates the performance of credit risk assessment models by measuring the average squared difference between the estimated and actual values. Lower MSE values indicate better model performance. The mathematical formulation of MSE is as follows:
  (19) $$\mathrm{MSE}=\frac{1}{n}\sum_{i=1}^{n}{\left(y_{i}-{\hat{y}}_{i}\right)}^{2}$$
  where *n* is the number of observations, $y_{i}$ represents the actual values, and ${\hat{y}}_{i}$ represents the predicted values.
- **Root Mean Squared Error (RMSE)**: Found in studies [13,22], it measures the square root of the average of squared differences between prediction and actual observation. RMSE is calculated as:
  (20) $$\mathrm{RMSE}=\sqrt{\frac{1}{n}\sum_{i=1}^{n}{\left({\hat{y}}_{i}-y_{i}\right)}^{2}}$$
  where ${\hat{y}}_{i}$ is the predicted value, $y_{i}$ is the actual value, and *n* is the total number of observations.
- **Root Relative Squared Error (RRSE)**: Mentioned in [22], RRSE measures the square root of the sum of the squared differences between predicted and actual values, normalized by the sum of the squared differences between the actual values and their mean. It offers a relative measure of the model prediction error. The formulation is:
  (21) $$\mathrm{RRSE}=\sqrt{\frac{\sum_{i=1}^{n}{\left(y_{i}-{\hat{y}}_{i}\right)}^{2}}{\sum_{i=1}^{n}{\left(y_{i}-\bar{y}\right)}^{2}}}$$
  where: *n* is the number of observations, $y_{i}$ represents the actual values, ${\hat{y}}_{i}$ represents the predicted values and $\bar{y}$ represents the mean of the actual values.
- **Relative absolute error (RAE)**: Reported in [22], RAE provides a relative measure of the average absolute error by comparing the sum of the absolute differences between predicted and actual values to the sum of the absolute differences between the actual values and their mean. The formulation is:
  (22) $$\mathrm{RAE}=\frac{\sum_{i=1}^{n}\left|y_{i}-{\hat{y}}_{i}\right|}{\sum_{i=1}^{n}\left|y_{i}-\bar{y}\right|}$$
  where: *n* is the number of observations, $y_{i}$ represents the actual values, ${\hat{y}}_{i}$ represents the predicted values and $\bar{y}$ represents the mean of the actual values.
- **Log Loss**: Mentioned in [27], Log Loss measures the performance of a classification model where the prediction is a probability value between 0 and 1. It penalizes false classifications and provides a better sense of the model’s uncertainty in its predictions.
  (23) $$\mathrm{Log}\;\mathrm{Loss}=-\frac{1}{n}\sum_{i=1}^{n}\left(y_{i}\log\left({\hat{y}}_{i}\right)+\left(1-y_{i}\right)\log\left(1-{\hat{y}}_{i}\right)\right)$$
  where ${\hat{y}}_{i}$ is the predicted probability of the positive class, $y_{i}$ is the actual class label (0 or 1), and *n* is the number of observations.
- **Brier Score**: Found in [26], it measures the accuracy of probabilistic predictions. Brier score has the following formulation:
  (24) $$BS=\frac{1}{N}\sum_{i=1}^{N}{\left(y^{\left(i\right)}-p\left(x^{\left(i\right)}\right)\right)}^{2}$$
  where $y^{\left(i\right)}$ is the label of the *i*-th example, $p(x^{\left(i\right)})$ denotes the probability of the *i*-th example classified into the positive class, and *N* is the total number of examples.

##### **Metaheuristics Metrics:** 

From the reviewed documents, we identified new metrics that can be added to the Metaheuristics Metrics category. These metrics provide deeper insights into credit risk assessment’s stability, feasibility, and computational efficiency. They help in understanding these methods’ robustness and practical applicability in various scenarios.

- **Stability**: Mentioned in [15], it assesses the stability of a feature selection algorithm against sampling fluctuations. The measure used is pairwise similarity (Jaccard Index) across all feature selection subsets $J_{m}$.
  (25) $$\mathrm{Stability}=\frac{2\sum_{m=1}^{M-1}\sum_{r=m+1}^{M}\mathrm{Sim}\left(J_{m},J_{r}\right)}{M(M-1)}$$
  where: *M* is the number of feature selection subsets, $\mathrm{Sim}(J_{m},J_{r})$ is the similarity measure (Jaccard Index) between feature selection subsets $J_{m}$ and $J_{r}$ formulated as $\mathrm{Sim}\left(J_{m},J_{r}\right)=\frac{|J_{m}\cap J_{r}|}{|J_{m}\cup J_{r}|}$. Because the scaled measure ranges between 0 and 1, it is possible to compare the stability of classifiers of various types.
- **Jaccard Stability Measure**: Described in [12], this metric compares the stability of feature selection algorithms. Jaccard stability measure is an intersection-based metric that finds the average similarity between different feature sets. The value of JSM ranges from 0 to 1, where a value near 1 is desirable as it means that the feature set selected does not change significantly and, hence, is more stable concerning small variations in the dataset. Formally, the Jaccard stability measure is calculated as:
  (26) $$\mathrm{JSM}=\frac{2}{Q(Q-1)}\sum_{q=1}^{Q-1}\sum_{q^{\prime }=q+1}^{Q}\frac{|S_{q}\cap S_{q^{\prime }}|}{|S_{q}\cup S_{q^{\prime }}|}$$
  where *Q* is the number of sub-samples of training data, q=1,…,Q. $S_{q}$ and $S_{q^{\prime }}$ denote the feature sets, $|S_{q}\cap S_{q^{\prime }}|$ denotes the number of common features.
- **Computational period**: Reported in [17], this metric represents the time taken to classify a loan as approved or rejected. It is expressed as follows:
  (27) $$\mathrm{Computational}\;\mathrm{period}\left(C_{t}\right)=\frac{L^{\prime }\times CPI}{R^{\prime }}$$
  where $L^{\prime }$ represents the count of loans, CPI is Cycles Per Instructions, and $R^{\prime }$ denotes the computational period.
- **Feasibility**: Reported in [14], this metric evaluates the practicality of implementing an algorithm in real-world scenarios. It is important to note that no mathematical description of the Feasibility metric is provided in the article.

##### **Feature Metrics:** 

In the reviewed documents, we identified a new metric that can be added to the Feature Metrics category. This metric focuses on the importance and stability of features used in machine learning models.

- **Shapley Values (SV)**: Mentioned in [10], seeks to determine the importance of each attribute in the prediction made by the model for a particular instance [71].

##### **Statistical Test Metrics:** 

In the reviewed documents, we identified new metrics that can be added to the Statistical Test Metrics category. These metrics enhance our ability to perform rigorous statistical analyses, ensuring that the observed differences in model performance are statistically significant. They provide robust methods for validating the reliability of experimental results and model comparisons.

- **Paired** ***t*****-test**: Reported in studies [17,28], it compares the means of two related groups.
- **Welch’s** ***t*****-test**: Used in [15], it compares the means of two independent groups while accounting for unequal variances and sample sizes, offering a more robust alternative to the standard *t*-test in heterogeneous data scenarios.
- **H-Measure**: Reported in [26], it provides an alternative to the AUC for evaluating model performance. The H-Measure is defined in [72].

### 3.7. Reported Algorithm Performance by Benchmark Dataset

This section summarizes the performance metrics of machine learning and metaheuristic-enhanced models as reported in the reviewed studies, organized by benchmark dataset. In line with the scope defined in Section 3.5 (Datasets and Variables) and Section 3.6 (Evaluation Metrics), this synthesis focuses exclusively on publicly available benchmark datasets and considers only classification performance metrics—such as accuracy, F1-score, AUC, and recall. Metrics related to statistical tests, computational cost, or feature selection (e.g., *t*-test, computational time, number of features selected) are excluded from this section, as they pertain to different analytical dimensions.

Performance results are reported only for benchmark datasets because these datasets are publicly available, well-documented, and frequently reused across multiple studies, enabling a more consistent and traceable synthesis of findings. In contrast, real-world application datasets are often proprietary, anonymized, or insufficiently described, limiting their comparability and the standardization of reported results.

Due to substantial heterogeneity in evaluation protocols—including differences in preprocessing steps, data balancing techniques, and metric choices—this summary does not attempt to standardize or directly compare results across studies. Rather, it highlights the variety of evaluation practices and provides contextual insights into model performance as reported by the original authors.

Table 8, organized by benchmark dataset, lists the studies that utilized each dataset, the algorithm(s) evaluated, the reported performance metric(s), and the corresponding values extracted from the original publications. The algorithm or model selected for inclusion in the table corresponds to the configuration that achieved the best performance in terms of accuracy within each respective study. This decision was necessary because authors often evaluate multiple configurations or classifiers and present disaggregated results. Selecting the most accurate configuration allows for a consistent and fair representation of each study’s most successful approach.

Cells without numeric values indicate that the respective metric was not reported by the original authors for that dataset. As discussed in Section 3.6, not all metrics were uniformly applied across all studies or datasets. Cells marked with an asterisk (*) indicate that no global performance metric was reported for the full model configuration—these cases typically involve multi-stage frameworks where evaluation is conducted at the component level.

Three specific cases deserve further clarification:

- Ref. [16] propose a two-stage selective learning framework, combining logistic regression for easy instances and a neural network for hard cases. However, they do not provide a unified performance metric that captures the behavior of the complete system; hence, no global value is included in the table.
- Similarly, ref. [24] introduce a two-stage rule extraction method, but only report performance metrics separately for the local rule extraction stage (see Table 6 in their paper) and the global optimization stage (see Table 7 in the same paper), without an aggregate evaluation of the integrated model.
- Ref. [20] report results for multiple classifiers and include both global (i.e., dataset-level) metrics and class-specific metrics. Specifically, accuracy and AUC are reported as overall summary measures, while precision, recall, and F1-score are provided separately for each class. As a result, only accuracy and AUC are included in the benchmark table, as the remaining metrics cannot be directly compared due to the lack of aggregated or averaged values.

### 3.8. Summary of Findings

This section synthesizes the findings from the literature review, addressing the five research questions that guided our investigation of the feature selection problem in credit risk assessment using machine learning and optimization techniques.

Firstly, we explored the various machine learning techniques employed in credit risk assessment (RQ1). Our review identified a range of methods, including logistic regression, decision trees, random forests, support vector machines, and neural networks. These techniques are extensively used for their ability to handle large datasets and complex relationships among variables, providing sophisticated tools for predicting borrower behaviour.

Secondly, we examined the feature selection methods used in credit risk assessment models (RQ2). Feature selection is a crucial step for enhancing model performance and interpretability. The reviewed studies highlighted a variety of methods, such as filter methods (e.g., mutual information, chi-squared test), wrapper methods (e.g., recursive feature elimination), and embedded methods (e.g., LASSO and tree-based methods). These techniques help identify the most relevant features that contribute to accurate credit risk predictions.

Thirdly, we investigated the application of optimization techniques in credit risk assessment (RQ3). Optimization techniques, including genetic algorithms, particle swarm optimization, and firefly algorithms, are used to fine-tune model parameters and improve the overall performance of credit risk models. These metaheuristic methods are essential for achieving optimal solutions in complex, high-dimensional data environments.

Fourthly, we identified the datasets and variables commonly used in credit risk assessment studies (RQ4). Public datasets such as the UCI Repository and Kaggle are frequently used for benchmarking models, while proprietary datasets from financial institutions provide rich, real-world data for model validation. Key variables include demographic information, financial status, credit history, and loan specifics, all critical for building robust credit risk models.

Lastly, we reviewed the evaluation metrics employed to assess the performance of credit risk assessment models (RQ5). Typical metrics include accuracy, precision, recall, F1-score, and area under the receiver operating characteristic curve (AUC-ROC). These metrics provide a comprehensive assessment of model effectiveness, ensuring the predictions are reliable and actionable.

In summary, our SLR highlights the significant advancements in credit risk assessment driven by machine learning and optimization techniques. Focusing exclusively on individual credit risk assessment, our work provides a detailed and targeted analysis that addresses the specific challenges and opportunities in this domain. The findings underscore the importance of feature selection and optimization methods in enhancing model performance and offer valuable insights for future research and practical applications in credit risk prediction.

## 4. Conclusions

This SLR has examined the methodologies and techniques used in credit risk assessment, highlighting key insights: the integration of machine learning models, the importance of feature selection methods, and the application of optimization techniques. The review provides a detailed understanding of how these approaches enhance the accuracy and efficiency of credit risk predictions. By focusing exclusively on individual credit risk, this review fills a significant gap in the literature, offering valuable guidance for researchers and practitioners.

Although this review does not conduct a direct performance comparison between algorithms, it includes a structured synthesis of performance metrics as reported in the reviewed studies, focusing on benchmark datasets. This additional layer of analysis contributes to a more transparent understanding of how different machine learning models have been evaluated under diverse conditions. It may also serve as a valuable reference for future benchmarking efforts and for identifying commonly adopted evaluation practices in the field.

1. Machine Learning Techniques: A wide array of supervised learning algorithms such as Logistic Regression, Decision Trees, Random Forest, Support Vector Machines, and Gradient Boosting Machines are predominantly used. Unsupervised learning, reinforcement learning, and deep learning models also contribute significantly to improving predictive accuracy.
2. Feature Selection Methods: Effective feature selection methods, categorized into filter, wrapper, and embedded approaches, are critical for enhancing model performance. Techniques like mutual information, chi-squared tests, Recursive Feature Elimination, LASSO, and tree-based methods are widely utilized.
3. Applications in Various Financial Contexts: Machine learning and optimization techniques are applied in traditional banking, peer-to-peer lending, and educational institutions, each with unique challenges and benefits. These applications improve the accuracy and efficiency of credit risk assessments and support financial planning and risk management.
4. Datasets and Variables: Public and proprietary datasets, including the Statlog (German Credit Data), Lending Club Loan Data, and Default of Credit Card Clients, are commonly used. Key variables include demographic information, financial status, loan specifics, credit history, employment details, and educational background.
5. Evaluation Metrics: A comprehensive set of metrics, such as accuracy, precision, recall, F1-score, AUC, MAE, and RMSE, are used to evaluate model performance. These metrics ensure a thorough model accuracy, efficiency, and practicality assessment.

### 4.1. Practical Recommendations

The practical recommendations derived from this review are intended to support practitioners in the implementation of effective credit risk assessment strategies. First, it is advisable to adopt advanced machine learning models, such as neural networks, support vector machines, and ensemble methods. These techniques have demonstrated superior predictive capabilities and can significantly enhance the accuracy of credit risk prediction models.

Second, practitioners should emphasize the importance of robust feature selection. Implementing methods from the filter, wrapper, and embedded families can help identify the most relevant variables influencing credit risk, thereby improving both the interpretability and overall performance of the models.

Third, the application of optimization techniques—particularly metaheuristic algorithms such as genetic algorithms, particle swarm optimization, and firefly algorithms—should be considered. These approaches can effectively reduce the dimensionality of the feature space and fine-tune model parameters, leading to improved performance, especially in complex data environments.

Additionally, it is essential to focus on data quality. High-quality, comprehensive datasets containing demographic information, financial status, credit history, and loan details are crucial for developing robust models. The reporting of datasets should be accompanied by a clear description of preprocessing steps to ensure reproducibility and clarity.

Finally, model performance should be rigorously evaluated using a diverse set of metrics. Metrics such as accuracy, precision, recall, F1-score, and AUC-ROC should be employed to provide a comprehensive assessment of model effectiveness. Ideally, at least one metric from each category should be reported to offer a balanced evaluation.

### 4.2. Future Research Directions

Future research in the field of individual credit risk assessment should aim to deepen and broaden the current understanding by addressing several key areas. The data sets used are characterized by being static and used for benchmarking. Incorporating real-time data into credit rating systems is another avenue with great potential. Integrating real-time or transactional data sources could facilitate dynamic risk assessment, providing institutions with timely information that facilitates proactive decision-making. Considering all financial products and institutions, it is essential to expand the application of credit risk assessment models beyond traditional banking. Research should examine how these models can be effectively implemented in organizational settings such as universities or government institutions that offer student loans or microenterprise loans. This would allow for more tailored and contextualized risk management frameworks.

Furthermore, future studies should seek to expand evaluation frameworks beyond conventional metrics. While accuracy, precision, recall, and AUC-ROC remain valuable, incorporating metrics that assess economic utility, business impact, and model robustness under varying conditions would provide a more comprehensive assessment of practical effectiveness. Furthermore, algorithm efficiency is a critical aspect; therefore, incorporating performance metrics such as computational cost or algorithmic complexity will further strengthen the research.

Collaboration between metaheuristic algorithms and machine learning represents another opportunity for future research. The high dimensionality and nonlinearity of financial datasets makes machine learning algorithms very sensitive to small variations, leading to errors. Solving the feature problem or optimizing the hyperparameters of machine learning algorithms are clear applications where further contributions can be made. This line of research could open the door to testing new advances in optimization techniques such as hybrid metaheuristics, self-adaptive metaheuristics, chaotic metaheuristics, and quantum metaheuristics.

Moreover, future reviews may benefit from conducting meta-analyses of predictive performance to quantitatively compare techniques under standardized conditions.

Lastly, ethical and fairness considerations should be at the forefront of future developments. As machine learning models are increasingly used to inform financial decisions, ensuring that these systems operate equitably and without bias is paramount. Research must address fairness constraints, transparency requirements, and the societal implications of automated credit decision systems.

### 4.3. Limitations

Although this systematic review was conducted following the PRISMA framework and established SLR guidelines, several limitations must be acknowledged.

First, the review was limited to studies published between 2019 and 2023, written in English, and indexed in SCOPUS and Web of Science. Relevant research from other time periods, languages, or databases—such as IEEE Xplore or Google Scholar—may have been excluded. However, this choice was justified based on the quality and scope of the selected databases.

Second, although we extracted and synthesized performance metrics reported in the reviewed studies (particularly for benchmark datasets), we did not conduct a quantitative meta-analysis or comparative evaluation under standardized conditions. The substantial heterogeneity in datasets, preprocessing steps, feature selection methods, and evaluation protocols across studies prevented direct comparison.

Third, while some studies addressed class imbalance using techniques such as SMOTE, the review does not systematically analyze the impact of these balancing methods on performance metrics like recall or F1-score for the minority class.

Fourth, the review does not assess underlying statistical assumptions such as data normality, feature independence, or potential multicollinearity, which could influence the interpretation of the models’ results. Additionally, while we discussed metaheuristic optimization techniques, we did not analyze computational costs or scalability of hybrid models on large datasets in depth.

These limitations should be considered when interpreting the findings and recommendations. Nonetheless, the systematic extraction and synthesis of data provide valuable insights into the current state of research on individual credit risk assessment using machine learning.

In conclusion, this systematic review has highlighted key trends and technological advancements in credit risk assessment. The outlined future research directions offer a roadmap for continued innovation, with the potential to improve predictive accuracy, operational relevance, and fairness in financial risk management.

## Figures and Tables

**Table 1:** Comparison of Contributions by Review.

Review	ICRA	ML	FS	OT	D&V	EM	EN-PPA	EN-AIT
[2]	✓	✓	✓	✓	✓	✓		
[6]	✓	✓		✓	✓	✓	✓	
[3]	✓	✓	✓	✓	✓	✓	✓	
[5]	✓	✓	✓	✓	✓	✓	✓	
[4]	✓	✓			✓	✓		
**Our work**	✓	✓	✓	✓	✓	✓	✓	✓

**Table 2:** Research Questions and Their Purposes.

ID	Question	Purpose
**RQ1**	What machine learning techniques are employed in individual credit risk assessment, and how are they applied in this context?	Identify and categorize machine learning algorithms for individual credit risk assessment and investigate their applications.
**RQ2**	Which feature selection methods are used in credit risk assessment models for individuals?	Explore techniques for selecting relevant features in individual credit risk assessment models.
**RQ3**	How are optimization techniques applied in the context of individual credit risk assessment?	Investigate the applications of optimization techniques specifically for individual credit risk assessment.
**RQ4**	What datasets and variables are commonly used in individual credit risk assessment studies?	Identify the typical data sources and key variables used specifically in individual credit risk assessment studies.
**RQ5**	What evaluation metrics are used to assess the performance of credit risk assessment models for individuals?	Examine the criteria used for evaluating the effectiveness of individual credit risk assessment models.

**Table 3:** Documents by Journal Title.

Journal Title	Documents
Expert Systems with Applications	[9,10,11]
Applied Soft Computing	[12,13]
Wireless Communications and Mobile Computing	[14]
SN Computer Science	[15]
Research in International Business and Finance	[16]
Multimedia Tools and Applications	[17]
Journal of Risk and Financial Management	[18]
Journal of Computational and Applied Mathematics	[19]
Journal of Applied Mathematics Statistics and Informatics	[20]
International Journal of Information Technology (Singapore)	[21]
Intelligent Systems in Accounting, Finance and Management	[22]
Innovations in Systems and Software Engineering	[23]
Information Sciences	[24]
Fuzzy Information and Engineering	[25]
Entropy	[26]
EAI Endorsed Transactions on Scalable Information Systems	[27]
Computers and Operations Research	[28]
Computers and Education: Artificial Intelligence	[29]
Computational Intelligence and Neuroscience	[30]
Annals of Emerging Technologies in Computing	[31]

**Table 4:** Citation Report of Reviewed Documents.

Document	Cited by	Document	Cited by
[12]	113	[16]	4
[9]	54	[15]	4
[19]	27	[27]	3
[11]	26	[14]	3
[13]	19	[10]	3
[28]	16	[23]	3
[24]	15	[30]	2
[22]	14	[29]	1
[18]	11	[31]	1
[21]	10	[20]	1
[26]	7	[17]	0
[25]	5		

**Table 5:** Public datasets (benchmarks).

Dataset Name	Source	Intances	Features	Labels
Statlog (Australian Credit Approval)	UCI	690	14	2
Statlog (German Credit Data)	UCI	1000	20	2
Default of Credit Card Clients	UCI	30,000	23	2
South German Credit Dataset	UCI	1000	21	2
Credit risk dataset	Kaggle	239	11	2
Kaggle’s Bank Loan Status dataset	Kaggle	12,535	19	2
Loan default prediction dataset	Kaggle	105,471	769	2
Give me some credit dataset	Kaggle	120,969	10	2
Credit card econometrics	Kaggle	1320	12	2
Kaggle Home Credit Default Risk	Kaggle	307,511	122	2
Czech Financial Dataset	Financial PKDD’99 Discovery Challenge	682	55	2
Thomas dataset	Credit Scoring and Its Applications, Book	1225	14	2
China UnionPay credit dataset	China UnionPay	11,017	199	2

**Table 6:** Real-world applications datasets.

Dataset Name	Source	Intances	Features	Labels
Lending Club loan data	Lendig club	n.a.	n.a.	n.a.
Lending club dataset	Lendig lub	42,538	143	2
Credit risk assessment data	Anonymous local bank in China.	10,744	10	n.a.
LendingClub (LC)	Lendig club	500,000	120	n.a.
Commercial Bank Credit Records	Anonymous commercial bank in China	27,520	27	5
Business credit score dataset	Data from a Latin American bank	20,835	585	2
Personal credit score	Data from a Latin American bank	76,209	936	2
General Data Protection Regulation (GDPR)	Commercial banks and savings institutions	1,000,000	n.a.	n.a.
Advanced Analytics of Credit Registry Dataset	undetermined	n.a.	n.a.	n.a.
WIND Dataset	Commercial bank’s personal credit database	n.a.	n.a.	n.a.
Croatian Bank Credit Risk Dataset 2009–2013	Large Croatian Bank	870,710	109	2
Croatian Bank Credit Risk Dataset 2004–2018	Large Croatian Bank	782,875	108	2
North Macedonia Credit Registry Data	Central Bank of the Republic of North Macedonia	1,000,000,000	52	5
Tunisian Bank Loan Data	Data from several Tunisian banks	n.a.	9	2
Lending Club (LC) dataset 2017–2018	Lendig club	477,131	16	2
Bank credit risk data	Comercial Banks	360	n.a.	n.a.
National Student Loans Dataset	University in Beijing, China	18,000	20	2
Student Payment Behavior Dataset	Private university in Peru	8495	13	2

**Table 7:** Confusion matrix for binary classification.

Confusion Matrix	Actual Positive (P)	Actual Negative (N)
**Predicted Positive (P)**	TP	FN
**Predicted Negative (N)**	FP	TN

**Table 8:** Summary of Reported Performance Metrics for Proposed Algorithms on Benchmark Datasets.

Dataset	Article	Algorithm Proposed	Accuracy	Recall	Precision	F1-Score	AUC-ROC	AUC-PRC
Statlog (German Credit Data) [54]	[12]	BS-RF	0.8400				0.7130	
	[15]	RFE-RF	0.7710	0.9061	0.7987	0.8490		
	[24]	Two-Stage Rule Extraction Method		*	*		*	
	[22]	LGBBO-RuleMiner	0.8930	0.7640	0.7510	0.7440	0.7910	0.8100
	[25]	HFA-FKNN	0.8714	0.8805	0.8918	0.8861		
Statlog (Australian Credit Approval) [55]	[24]	Two-Stage Rule Extraction Method		*	*		*	
	[22]	LGBBO-RuleMiner	0.8700	0.8700	0.8690	0.8690	0.9270	0.9180
Default of Credit Card Clients [61]	[16]	Two-stage Selective Learning Framework					*	
	[21]	Optimized Decision Tree	0.8220	0.6900	0.3500	0.4125	0.7000	
South German Credit Dataset [56]	[25]	HFA-RF	0.8621	0.8677	0.8700	0.8631		
Thomas Dataset [1]	[25]	HFA-RF	0.8304	0.8269	0.8371	0.8314		
Kaggle’s Bank Loan Status dataset [58]	[12]	BS-RF	0.7310				0.7310	
Credit Card Econometrics [64]	[25]	HFA-RF	0.9902	1.0000	0.9805	0.9901		
China UnionPay Credit Dataset [65]	[28]	WGAN + KPLS-QPSO-HFS		0.6202			0.6309	
Give Me Some Credit Dataset [60]	[16]	Two-stage Selective Learning Framework					*	
Kaggle Home Credit Default Risk [59]	[20]	XGBoost (Tuned)	0.7600	*	*	*	0.7391	
Credit risk dataset [63]	[17]	ABSMPNN	0.9890	0.9910	0.9904	0.9865	0.9800	
Loan default prediction dataset [57]	[28]	WGAN + KPLS-QPSO-HFS		0.5971			0.6375	
Czech Financial Dataset [62]	[31]	CSM–EWD-SVM	0.8670		0.9960			

Note: Cells marked with an asterisk (*) indicate that no global performance metric was reported for the full model configuration in the original article. These cases typically involve multi-stage frameworks where evaluation is conducted at the component level.

## Data Availability

The raw data supporting the conclusions of this article will be made available by the authors on request.
